# Plants maximise chloride uptake during early vegetative development to stimulate cell expansion, maturation of the photosynthetic apparatus, and growth

**DOI:** 10.1111/tpj.70378

**Published:** 2025-08-30

**Authors:** Procopio Peinado‐Torrubia, Juan D. Franco‐Navarro, Marta Lucas, David Romero‐Jiménez, Francisco J. Moreno‐Racero, Pablo Díaz‐Rueda, Miguel A. Rosales, Marika Lindahl, Antonio Díaz‐Espejo, Rosario Álvarez, José Manuel Colmenero‐Flores

**Affiliations:** ^1^ Instituto de Recursos Naturales y Agrobiología Consejo Superior de Investigaciones Científicas (CSIC) Seville 41012 Spain; ^2^ Instituto de Bioquímica Vegetal y Fotosíntesis Consejo Superior de Investigaciones Cientificas (CSIC) and Universidad de Sevilla Seville 41092 Spain; ^3^ Departamento de Biología Vegetal y Ecología, Faculty of Biology Universidad de Sevilla Seville 41012 Spain; ^4^ Present address: Hygiene Quality and R&D Department, CLECE S.A. University Hospital of Puerto Real (HUPR) Cádiz 11510 Spain; ^5^ Present address: Estación Experimental del Zaidín Consejo Superior de Investigaciones Científicas (CSIC) Granada 18008 Spain

**Keywords:** chloride, plant nutrition, beneficial macronutrient, early vegetative development, nitrate, morphogenesis, chloroplast, photosynthesis, photosystem II, electron transport rate

## Abstract

Despite being an essential micronutrient and its recent classification as a beneficial macronutrient, chloride (Cl^−^) has traditionally been considered of limited agricultural relevance and a potentially toxic saline ion. This study provides the first comprehensive demonstration of the quantitative and qualitative importance of Cl^−^ during early vegetative development (EVD) of tobacco and *Arabidopsis thaliana* plants. During this developmental stage, these and other species (including celery, lettuce, Swiss chard, spinach, squash, tomato, chili pepper, eggplant, and perennial ryegrass) exhibit the highest demand and transport rate of this non‐assimilable mineral nutrient to maximise growth of these herbaceous and also woody (such as citrus and olive) species. While Cl^−^ promotes cell expansion across all growth stages, its particularly pronounced stimulation of plant growth during EVD is associated with enhanced photosynthetic performance and PSII activity. This enhancement is in turn linked to a reduction in non‐regulated energy dissipation in PSII and an increase in the electron transport rate, along with ultrastructural changes in chloroplasts, underscoring that Cl^−^ is specifically required during EVD to drive the maturation of the photosynthetic apparatus. Unlike adult plants, the growth deficiencies caused by sub‐macronutrient Cl^−^ levels during EVD cannot be mitigated by equivalent nitrate (NO_3_^−^) supplementation. As EVD concludes, plant demand for Cl^−^ gradually decreases, accompanied by a reduced growth response to Cl^−^ and an increased reliance on NO_3_^−^, emphasising stage‐specific nutrient needs. The relevance of Cl^−^ as a morphogenic driver during a critical stage of development has significant implications for optimizing agronomic practices, particularly by reducing dependence on nitrogen fertilisers.

## Significance Statement

This study provides the first comprehensive evidence for the quantitative and qualitative importance of chloride during early vegetative development (EVD) in 15 herbaceous and woody species. We demonstrate that chloride acts as a morphogenic driver that mitigates mechanical and biochemical constraints in immature leaves, thereby enhancing PSII performance at a critical growth stage, while also highlighting shifting chloride versus nitrate requirements throughout plant development.

## INTRODUCTION

Chloride (Cl^−^) has often been regarded as a nutrient of low relevance in agriculture and even a toxic component in soil or irrigation water. Two recurring ideas still prevail in both scientific and agronomic fields (Xu et al., 2000; White & Broadley, 2001; Geilfus, 2018, 2019): (1) the small amount of Cl^−^ required by plants is readily available in nature, making Cl^−^ deficiency a rare occurrence; (2) excessive Cl^−^ accumulation in plants is a more common phenomenon, leading to detrimental effects in crop yield and quality. This negative perception arises from two widely recognised phenomena: the toxicity due to excessive Cl^−^ accumulation in sensitive crops under saline conditions (Brumós et al., 2010; Henderson et al., 2014; Li et al., 2017; Teakle & Tyerman, 2010; Colmenero‐Flores et al., 2020) and the longstanding belief in an antagonistic relationship between Cl^−^ and nitrate (NO_3_^−^), whereby Cl^−^ is thought to impair the plant's ability to take up and transport NO_3_^−^ (Bar et al., 1997; Britto et al., 2004; Siddiqi et al., 1990; Wege et al., 2017; Xu et al., 2000). Since NO_3_^−^ is the main source of nitrogen (N), and N is the main limiting factor for the growth of terrestrial plants, synthetic N fertilisers are excessively used in agriculture, while Cl^−^ is often considered an indicator of poor quality in fertilisers (European Parliament and Council of the European Union, 2019) and is frequently avoided.

However, Cl^−^ is one of the 14 essential mineral nutrients, performing different biochemical roles in plant cells (Cakmak et al., 2023). Chloride is crucial for stabilising the oxygen‐evolving complex (OEC) in Photosystem II (PSII), where two Cl^−^ ions specifically bind near the Mn_4_CaO_5_ cluster (Imaizumi & Ifuku, 2022). This has been the biochemical role proposed as truly essential for plants, which would barely require trace levels of Cl^−^ in the medium (Terry, 1977; Wege et al., 2017). Additionally, Cl^−^ acts as a cofactor in various enzymatic reactions, regulates cellular osmosis and turgor (e.g., in stomatal and motor cells for nastic movements), stabilises membrane electric potential, and maintains pH gradients (Broadley et al., 2012; Flowers, 1988; Hänsch & Mendel, 2009; White & Broadley, 2001; Xu et al., 2000). To accomplish these functions, it was generally believed that the minimum Cl^−^ requirement for adequate growth in most glycophyte species was within the range of 0.1–0.5 mg g^−1^dry weight (mg g^−^1 DW), which corresponds to the content of a micronutrient (Broadley et al., 2012; Broyer et al., 1954; Heckman, 2006; Johnson et al., 1957; White & Broadley, 2001; Xu et al., 2000).

Recently, Cl^−^ has also been classified as a beneficial macronutrient, as higher plants expend metabolic energy to accumulate Cl^−^ at much higher concentrations than the micronutrient level (about one to two orders of magnitude), performing roles that improve: cell growth and water balance (Franco‐Navarro et al., 2016); mesophyll CO_2_ diffusion conductance and water relations (Franco‐Navarro et al., 2019; Maron, 2019); and NO_3_^−^ assimilation (Lucas et al., 2024; Peinado‐Torrubia et al., 2023; Ramirez‐Builes et al., 2023; Rosales et al., 2020). These functions significantly increase water‐, nitrogen‐, and carbon/energy‐use efficiency—key aspects of plant nutrition—thus boosting biomass production (Cakmak et al., 2023; Colmenero‐Flores et al., 2019). Furthermore, Cl^−^ at macronutrient levels improves plant resistance to water deficit (Franco‐Navarro et al., 2021) and to low NO_3_^−^ availability (Lucas et al., 2024; Neocleous et al., 2021), improving plant resilience. As a proof of concept, Cl^−^ fertilisation is currently being tested in novel fertiliser formulations to enhance the cultivation of various agriculturally important species, including tobacco, tomato, lettuce, potato, coffee, etc. (Hütsch et al., 2024; Koch et al., 2021; Lucas et al., 2024; Mo et al., 2024; Neocleous et al., 2021; Neocleous et al., 2024; Pace et al., 2020; Ramirez‐Builes et al., 2023).

Chloride transport and accumulation can vary substantially among species and even among varieties within the same species (Brumós et al., 2010; Colmenero‐Flores et al., 2019; Rosales et al., 2020; White & Broadley, 2001). Such variation in Cl^−^ accumulation within the beneficial macronutrient range may reflect species‐specific physiological strategies, such as the role of turgor in maintaining leaf firmness (Yolcu et al., 2021). For example, leaf Cl^−^ concentrations in olive trees are around 10 mg g^−1^ DW, whereas in leafy vegetables such as chard, they can reach approximately 100 mg g^−1^ DW (Rosales et al., 2020). However, the extent to which these differential Cl^−^ levels reflect species‐ or variety‐specific requirements remains poorly understood and warrants further investigation.

Our studies characterising Cl^−^ as a beneficial macronutrient were conducted on adult plants during late vegetative and reproductive stages. We consistently observed throughout the course of these experiments that growth enhancement induced by Cl^−^ was apparently more pronounced during early vegetative development (EVD) than in later stages (unpublished data). EVD is a critical stage in plant establishment characterised by high cell division and elongation rates and incomplete development of chloroplasts and electron transport systems. Plant ontogeny (juvenile versus adult) and leaf age influence the efficiency with which the photosynthetic machinery is assembled and maintained. During early developmental stages (e.g., during EVD or in expanding leaves), photosynthetic capacity is limited by anatomical and physiological constraints. These include fewer and smaller chloroplasts, incomplete assembly of the photosynthetic apparatus, lower stomatal conductance and mesophyll conductance, reduced pigment levels, and diminished accumulation of photosynthetic proteins (Chondrogiannis & Grammatikopoulos, 2016; Pallardy, 2008; Pantin et al., 2012; Rawson et al., 1983; Zoschke et al., 2007). Collectively, we will refer to this as an immature photosynthetic machinery. Consequently, maximum quantum efficiency of PSII and net CO_2_ assimilation per unit leaf area remain low during early development. In Mediterranean species, overall plant age further influences photosynthetic maturation. For example, adult *Nerium oleander* exhibits higher mature leaf CO_2_ assimilation than juveniles, owing to greater electron flow to carboxylation and reduced photorespiration, while adult *Phlomis fruticosa* leaves show improved water‐use efficiency (Chondrogiannis & Grammatikopoulos, 2016). Recent research suggests that early steps in the *in vitro* photoassembly of PSII, including the OEC, require higher Cl^−^ concentrations than previously assumed (Russell & Vinyard, 2022; Vinyard et al., 2019). This led to the postulation that plants may require Cl^−^ at levels of a macronutrient to support maximum PSII‐OEC assembly rates and the optimal repairing of photodamaged thylakoids during thylakoid ontogeny (Raven, 2020).

Therefore, Cl^−^ combines properties related to the stimulation of cell expansion, and potentially, of the photosynthetic capacity, which would make it specifically more suitable for optimizing plant growth during early development. We hypothesized that plants, rather than NO_3_^−^, may specifically require increased demand and accumulation of Cl^−^ for optimal growth and development during EVD, and that its potential role in enhancing both cell expansion and the activity of the photosynthetic machinery may underlie this requirement.

In this study, we report that Cl^−^ at levels of a macronutrient is essential for the maximum growth of herbaceous (e.g., tobacco, *Arabidopsis thaliana* and tomato) and woody (e.g., citrus and olive) plant species during early development, and contrary to the plant adult stage, its deficiency cannot be mitigated by additional NO_3_^−^ supply. We further associated this requirement with enhanced cell expansion and maturation of the photosynthetic machinery, leading to significant improvements in PSII performance and photosynthetic electron transport rate (ETR). These responses are closely aligned with the peak demand for Cl^−^ during EVD, which gradually declines as plants enter into later developmental stages, where NO_3_^−^ nutrition becomes increasingly important and can restore growth in the absence of macronutrient Cl^−^.

## RESULTS

### Chloride acts as a growth‐limiting macronutrient during early plant development

**Figure 1:**
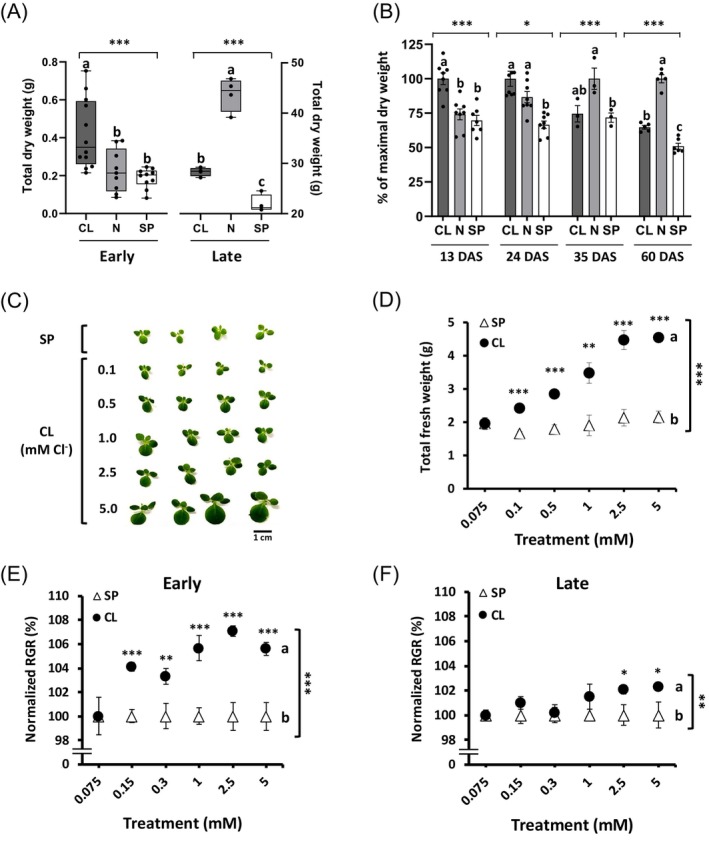
Chloride nutrition at macronutrient levels specifically promotes plant growth during early plant development. (A) Total dry weight of tobacco plants at early (13 days after sowing, DAS) and late development (65 DAS). Plants received a basal nutrient solution supplemented with one of the following: 5 mm Cl^**−**^ (CL), 5 mm NO_3_^**−**^ (N), or sulphate + phosphate (SP) salts, each providing identical cation concentrations. (B) Biomass growth (% of maximal dry biomass) of tobacco plants at different developmental stages. Days 13–35 represent various stages of vegetative development, while day 60 corresponds to the onset of reproductive development. (C) Tobacco plants at 13 DAS treated with increasing Cl^**−**^ concentrations, counterbalanced with SP salts to maintain identical cation concentrations. (D) Fresh weight of 21 DAS plants treated with increasing Cl^**−**^ concentrations, counterbalanced with SP salts. (E, F) Cl^**−**^ stimulation of the plant relative growth rate (RGR) during early vegetative development (EVD) versus late development. RGR was calculated within the 9–27 DAS interval for EVD and the 27–64 DAS interval for late development. Both datasets were normalised to the RGR of SP‐treated plants (mean ± SE, *n* = 6). Asterisks indicate statistically significant differences (one‐way ANOVA, ***P < 0.001, **P < 0.01, *P < 0.05). Different letters in panels E and F indicate statistically significant differences between treatments (MANOVA, *P* < 0.05).

During EVD, a strong growth‐promoting effect of Cl^−^ was observed in tobacco plants, achieving up to double the dry biomass compared with plants in the N and SP treatments (Figure 1A; Figure S1). Growth stimulation by Cl^−^ was also achieved during other early developmental stages such as germination (Figure S2a) and the establishment of etiolated seedlings (Figure S2b). Notably, deficiency symptoms observed in plants without the supplementation of Cl^−^ at levels of a macronutrient (SP plants) could not be mitigated by NO_3_^−^ supply (N plants), in contrast to findings in adult plants (Figure 1A; Figure S1; Franco‐Navarro et al., 2016, 2019). Thus, maximum growth during EVD required high Cl^−^ availability (5 mm Cl^−^ in the CL treatment), whereas adult plants achieved maximum growth with a high NO_3_^−^ supply (10 mm NO_3_^−^ in the N treatment) despite limited Cl^−^ availability (75 μm Cl^−^; Figure 1A and Figure S1). Therefore, when examining the dry biomass throughout the entire developmental cycle of tobacco plants, Cl^−^ was revealed as a critical growth promoter during EVD, while NO_3_^−^ was more influential in later developmental stages (Figure 1B). A clear association between Cl^−^ supply (0.1–5.0 mm Cl^−^) and biomass during EVD was found in tobacco plants 13 days after sowing (DAS; Figure 1C) and 21 DAS (Figure 1D). Consequently, the relative growth rate (RGR) showed significantly higher responsiveness to Cl^−^ supply during EVD (Figure 1E) compared with later developmental stages (Figure 1F).

The growth‐promoting role of Cl^−^ during EVD was further observed across other herbaceous and woody plant species. It was found by biomass increase in citrus (Figure S3a) and tomato (Figure S3b), while visual observation supported this for olive (Figure S3c) and lettuce (Figure S3d). As previously observed in tobacco plants, Cl^−^ was also specifically critical during early development in tomato plants, while NO_3_^−^ became increasingly relevant for promoting growth at later stages (Figure S3b). Thus, plants showed a strict Cl^−^ requirement to develop their maximum growth potential during EVD, while NO_3_^−^ was more relevant during subsequent development, being able to compensate for Cl^−^ deficiency as a macronutrient.

### Plants exhibit maximal chloride demand during early vegetative development

**Figure 2:**
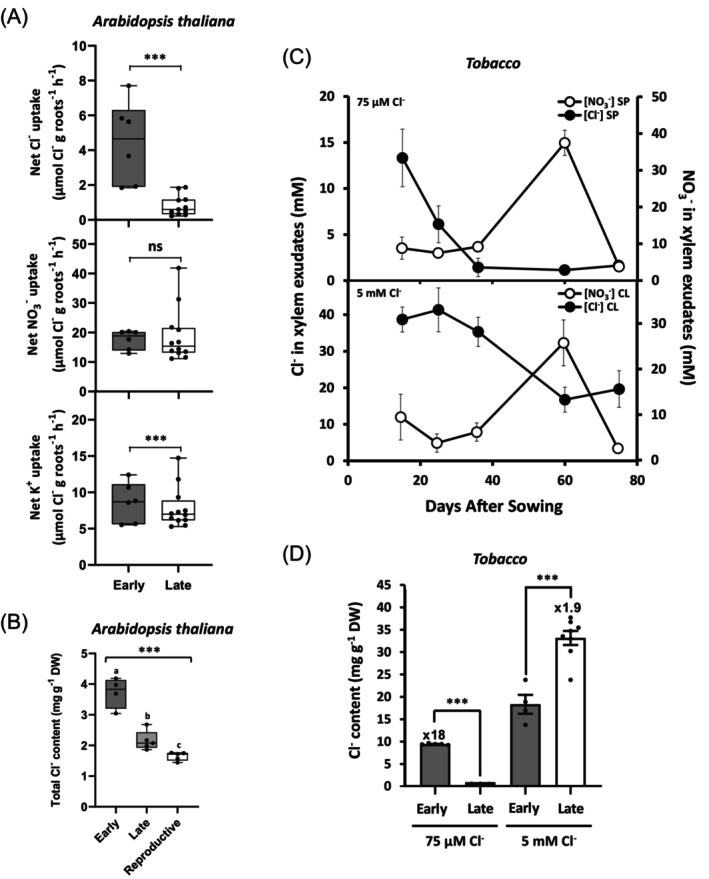
Cl^−^ demand peaks during early vegetative development and decreases progressively as plant development advances. (A) Net uptake rates of Cl^**−**^, NO_3_^**−**^, and K^+^ in *Arabidopsis thaliana* plants grown hydroponically during early vegetative development (15 DAS) and late vegetative development (30 DAS) under 5 mm Cl^**−**^ (CL treatment). (B) Cl^**−**^ accumulation in *Arabidopsis thaliana* plants grown hydroponically at different developmental stages: 20 DAS (early vegetative), 30 DAS (late vegetative), and 42 DAS (reproductive). Total plant Cl^**−**^ content was measured 12 h after supplying 5 mm Cl^**−**^ to plants previously grown in low‐Cl^**−**^ conditions (SP medium). (C) Cl^**−**^ and NO_3_^‐^ concentration in the xylem sap of tobacco plants throughout development, from early vegetative (15 DAS) to reproductive development (60 DAS, onset of flowering), under micronutrient (75 μm) and macronutrient (5 mm) Cl^**−**^ ranges. (D) Cl^**−**^ accumulation in the shoots of tobacco plants during early vegetative development (13 DAS) compared with late development (60 DAS) in plants treated with Cl^**−**^ at micronutrient (75 μm) and macronutrient (5 mm) levels. Data are presented as mean ± SE (*n* = 5–10). Asterisks indicate statistically significant differences (one‐way ANOVA, ***P < 0.001, ‘ns’ (not significant) *P* > 0.05). Different letters denote statistically significant differences between treatments (ANOVA, *P* < 0.05).

Given the specifically stronger growth‐promoting effect of Cl^−^ nutrition during EVD, we hypothesized that plants have a higher Cl^−^ requirement and thus display increased Cl^−^ demand during this stage. Due to limitations in cultivating tobacco plants hydroponically, we measured net Cl^−^ uptake in hydroponically grown *Arabidopsis thaliana* (Arabidopsis) plants. In plants treated with 5 mm Cl^−^, net Cl^−^ uptake was approximately five times higher during early versus late vegetative development, while net K^+^ and NO_3_^−^ uptake rates remained constant (Figure 2A). Twelve hours after applying 5 mm Cl^−^ to Arabidopsis plants grown under low‐Cl^−^ conditions (SP treatment), Cl^−^ accumulation peaked during EVD and progressively declined with plant maturity, decreasing around threefold by the reproductive stage (Figure 2B).

In tobacco, Cl^−^ demand was assessed by quantifying ion concentration in xylem sap across developmental stages. The highest Cl^−^ concentration in xylem sap occurred during EVD in plants treated with both micro‐(75 μm Cl^−^) and macronutrient (5 mm Cl^−^) supplements (Figure 2C). Notably, plants treated with 75 μm Cl^−^ showed a 10‐fold reduction in xylem sap Cl^−^ concentration between the early vegetative (15 DAS) and the late vegetative (35 DAS) and reproductive (60 DAS) stages. By contrast, NO_3_^−^ concentration in xylem sap peaked in reproductive stage plants (60 DAS), aligning with the onset of flowering (Figure 2C). During EVD, aerial organs of tobacco treated with 75 μm Cl^−^ contained 18 times more Cl^−^ than those of adult plants (Figure 2D). However, in plants treated with 5 mm Cl^−^, adult tissues contained double the Cl^−^ concentration compared with early vegetative tissues, as Cl^−^ accumulates over time when the external supply (5 mm Cl^−^) surpasses dilution capacity from growth, ultimately concentrating in large vacuolated cells of mature photosynthetic tissues (Colmenero‐Flores et al., 2019).

**Figure 3:**
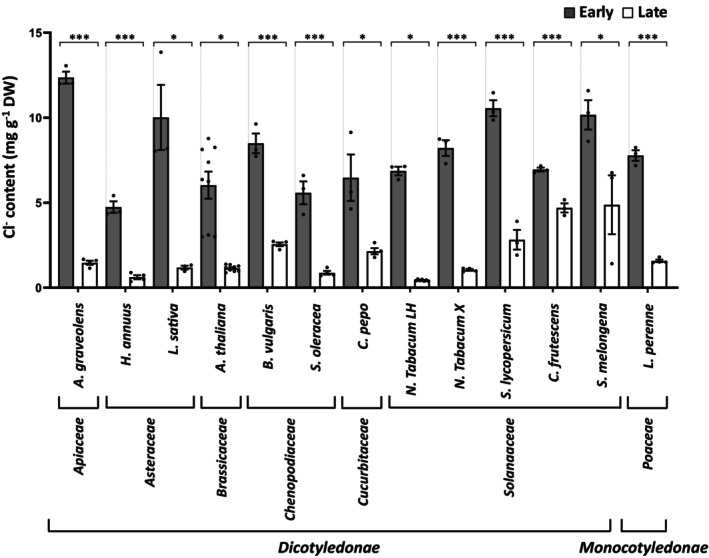
Peak Cl^−^ demand during early development is a general phenomenon in land plants. The following species, which also appear in Supplementary Table S1, are listed from left to right: *Apium graveolens*, *Helianthus annuus*, *Lactuca sativa*, *Arabidopsis thaliana* ecotype Columbia/Col‐0, *Beta vulgaris* var. cicla, *Spinacia oleracea*, *Cucurbita pepo*, *Nicotiana tabacum* var. light habana, *Nicotiana tabacum* var. xanthi, *Solanum lycopersicum* var. campbell33, *Capsicum frutescens*, *Solanum melongena*, and *Lolium perenne*. Cl^**−**^ accumulation during early vegetative development compared with late development stage in several land species treated with 75 μm Cl^**−**^. Asterisks indicate statistically significant (one‐way ANOVA, ***P < 0.001, *P < 0.05; mean ± SE, *n* = 4–6).

Given that external application of 75 μm Cl^−^ allows the phenomenon of higher Cl^−^ demand during EVD to be easily visualized in tobacco plants (Figure 2D), we applied this treatment to 13 other monocot and dicot species to establish whether this is a more general phenomenon in higher plants. The results obtained in Figure 3 show that the Cl^−^ content during EVD is much higher than during late development, reflecting that the maximum demand for Cl^−^ during early development is a widespread phenomenon across diverse plant taxa.

### Chloride improves cell expansion and photosynthetic parameters during EVD

**Figure 4:**
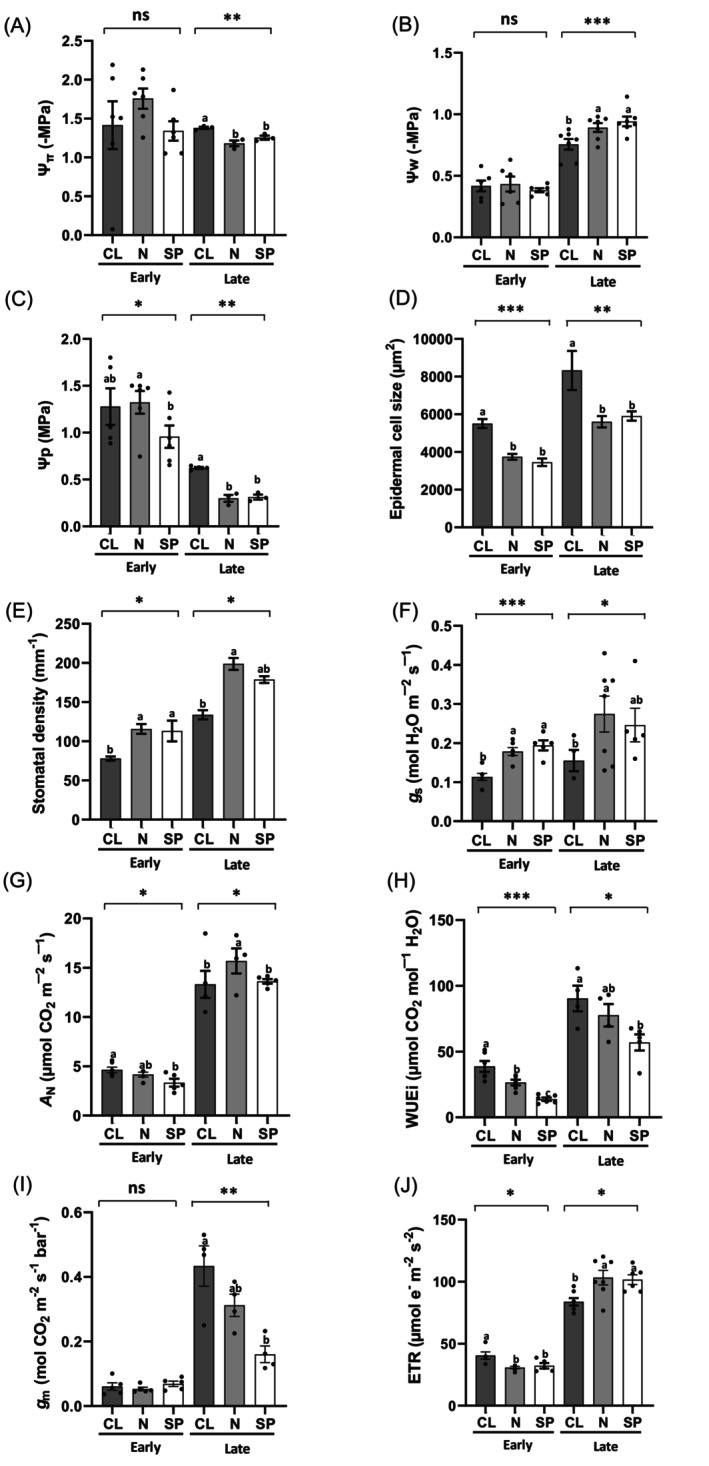
Physiological and morphological alterations driven by Cl^−^ nutrition in leaves of tobacco plants during early and late vegetative development. Tobacco plants were grown with a basal nutrient solution supplemented with either 5 mm Cl^**−**^ (CL), 5 mm NO_3_^**−**^ (N) or sulphate + phosphate (SP) salts containing identical concentrations of cations. Various physiological and morphological parameters were characterised: leaf osmotic potential (ψ_π_, A); leaf water potential (ψ_w_, B); leaf turgor pressure (ψ_p_, C); epidermal cell size (D), stomatal density (E), stomatal conductance (*g*_*s*_, F); net photosynthetic rate (*A*_N_, G); intrinsic water‐use efficiency (WUEi, H); mesophyll diffusion conductance to CO_2_ (*g*_*m*_, I); and electron transport rate (ETR, J). Measurements were conducted during early vegetative development (15 DAS) and late vegetative development (35 DAS). Cell water parameters (A–C) were measured in leaf discs from the primary leaf during early vegetative development and from the first fully expanded leaf during late vegetative development. Photosynthetic parameters (F, G, H, I, and J) were determined using an open gas exchange system with a fluorescence chamber. Asterisks indicate statistically significant differences (one‐way ANOVA, ***P < 0.001, **P < 0.01, *P < 0.05, ‘ns’ (not significant) *P* > 0.05; mean ± SE, with the number of biological and technical replicates shown in Material and Methods). Different letters indicate statistically significant differences (ANOVA, *P* < 0.05).

In adult plants, the beneficial roles of Cl^−^ are primarily associated with its osmoregulatory functions and its capacity to stimulate cell expansion, enhancing processes such as cell water balance, plant water relations, and nitrogen‐use efficiency (NUE) (Figure 4A–C; Colmenero‐Flores et al., 2019; Cakmak et al., 2023). During EVD, plants exhibited better water status compared with those in the late vegetative stage, characterised by comparable osmotic potential (ψ_π_; Figure 4A), less negative leaf water potential (ψ_w_; Figure 4B, indicating higher water content), and greater turgor (ψ_P_; Figure 4C). While the Cl^−^ treatment did not significantly alter these parameters during EVD (Figure 4A–C), it notably enhanced leaf cell expansion, resulting in larger epidermal cells (Figure 4D), including guard cells (Figure S4a). As occurs during late development, higher epidermal cell expansion contributed to a reduced stomatal density in Cl^−^‐treated plants (Figure 4E), which was accompanied by significantly lower stomatal conductance (*g*_s_) during EVD (Figure 4F).

During early developmental stages (e.g., during EVD or in expanding leaves), photosynthetic capacity is limited by anatomical and physiological constraints, including the incomplete development of chloroplasts and electron transport systems (see Introduction and references therein). Consistently, several photosynthetic parameters, including net photosynthetic rate (*A*_N_), intrinsic water‐use efficiency (WUEi), mesophyll diffusion conductance to CO_2_ (*g*_*m*_) and PSII electron transport rate (ETR), were strongly impaired in the primary leaves of tobacco plants during EVD compared with leaves of adult tobacco plants at later vegetative stages (Figure 4G–J, respectively). Among these parameters, *A*_*N*_, WUEi, and ETR were specifically enhanced by Cl^−^ during EVD. Notably, *A*_N_ was slightly stimulated by Cl^−^ during EVD and by NO_3_^−^ during late development (Figure 4G), while the Cl^−^ treatment significantly enhanced ETR during EVD, with no such effect observed in later vegetative stages (Figure 4J). It is expected that the simultaneous stimulation by Cl^−^ of ETR, *A*_N_, and WUEi provides a significant advantage for improving the growth capacity of C3 species during EVD. The ETR/*A*_N_ ratio provides an estimate of how many electrons transported through PSII are used per molecule of CO_2_ assimilated. During EVD, lower PSII efficiency determined higher ETR/*A*_N_ ratios, but Cl^−^ did not modify this parameter during either EVD or late development (Figure S4b).

### Chloride improves PSII performance during EVD

Recent findings suggest that Cl^−^ nutrition at levels of a macronutrient may be required to achieve the maximum rate of PSII assembly (Raven, 2020). Therefore, the improvement in ETR observed in Cl^−^‐treated plants likely stems from enhanced integrity of PSII. To test this hypothesis, we performed chlorophyll a fluorescence measurements with an IMAGING‐PAM system, which detects rapid changes in photochemical and non‐photochemical parameters of PSII with high resolution and sensitivity (see Methods). The effective quantum yield of PSII [Y(II)], the regulated energy dissipation of PSII [Y(NPQ)], and the non‐regulated energy dissipation of PSII [Y(NO)] were all significantly affected by the Cl^−^ treatment (Figure S5). Notably, Y(NO), which reflects the loss of PSII integrity due to factors like photodamage and inhibition of the repairing mechanisms, was significantly reduced in Cl^−^‐treated tobacco plants during both early and late vegetative development, pointing to higher protection or integrity of PSII (Figure S5).

**Figure 5:**
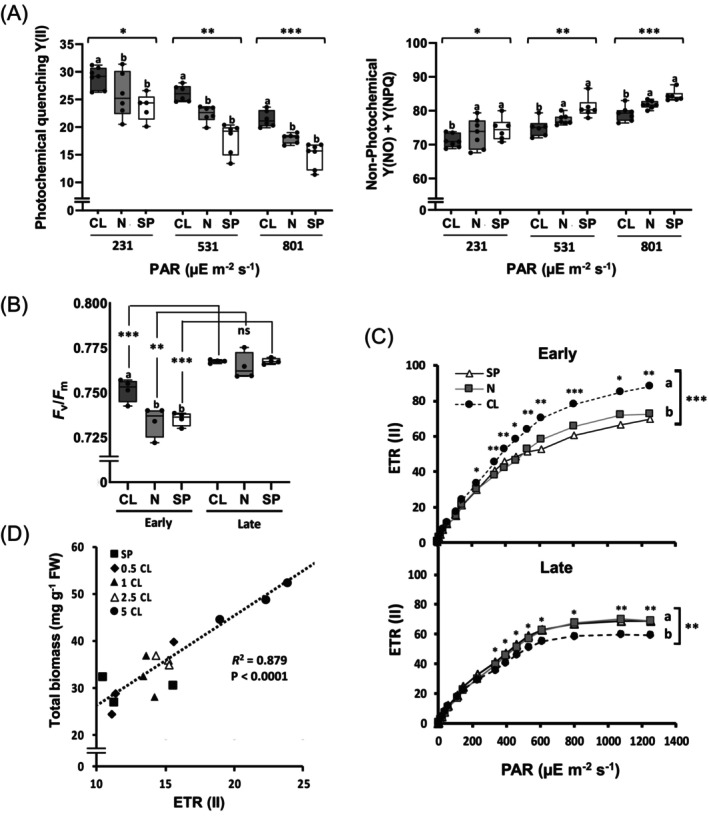
Regulation by Cl^−^ of the PSII performance during early and late vegetative development. Tobacco plants were grown with a basal nutrient solution supplemented with either 5 mm Cl^**−**^ (CL), 5 mm NO_3_^**−**^ (N), and sulphate + phosphate (SP) salts containing identical concentrations of cations and were evaluated during early (15 DAS) and late vegetative development (35 DAS). (A) PSII energy partitioning—photochemical efficiency [effective quantum yield, Y(II)] and non‐photochemical components [regulated energy dissipation, Y(NPQ), and non‐regulated energy dissipation, Y(NO)]—was determined in plants grown under different light intensities using an Imaging‐PAM fluorometer. (B) F_v_/F_m_ values were measured under the same conditions. (C) The CL treatment enhanced the electron transport rate [ETR(II)] during EVD but not during late vegetative development. (D) Correlation between ETR (II) and dry biomass during EVD in plants treated with increasing Cl^**−**^ concentrations. Asterisks indicate statistically significant differences determined through one‐way ANOVA, ***P < 0.001, **P < 0.01, *P < 0.05, ‘ns’ (not significant) *P* > 0.05 (mean ± SE, *n* = 4–6). Different letters denote statistically significant differences between treatments (in A and B panels, *P* < 0.05) and MANOVA tests (in C panel, *P* < 0.05).

Consistent with the lower photosynthetic capacity of tobacco plants during EVD observed in Figure 4G, Y(II) was lower (Figure S6a) and Y(NO) higher (Figure S6c) during EVD compared with adult plants, particularly across higher light intensities (531 and 801 PAR). Remarkably, the reduction in Y(II) was evident in N and SP plants but not in Cl^−^‐treated plants, where Y(II) during EVD was comparable to that of adult plants (Figure S6a). Thus, higher Cl^−^ availability during EVD significantly enhanced Y(II) across different light intensities (Figure 5A), indicating a greater proportion of absorbed light energy being utilised for photochemical processes and lower energy dissipation through non‐photochemical processes in PSII (Figure 5A), including Y(NPQ) and Y(NO). The maximum quantum yield (*F*_v_ */F*_m_ ratio), the most common measure of PSII efficiency, was notably lower during EVD compared with adult plants, reflecting the incomplete maturation of the photosynthetic machinery (Figure 5B). However, the Cl^−^ treatment significantly increased *F*_v_ */F*_m_ during EVD relative to the N and SP treatments (Figure 5B). Better PSII performance during EVD in Cl^−^‐treated plants was reflected in a higher efficiency of electron transport, ETR(II), suggesting an improved ability to sustain photochemical activity and mitigate photoinhibition under varying light conditions (Figure 5C). To further explore the relationship between Cl^−^ nutrition, ETR(II), and biomass growth during EVD, tobacco plants were exposed to increasing Cl^−^ concentrations, revealing a statistically significant correlation between Cl^−^ availability, the consequent increase in ETR(II), and the total biomass achieved by the plant (Figure 5D).

To further validate the positive impact of Cl^−^ nutrition on PSII performance during EVD, photosynthetic pigments were quantified, given the critical role of chlorophyll b in stabilising the major light‐harvesting complex (LHC) of PSII proteins (Plumley & Schmidt, 1995; Tanaka & Tanaka, 2011), where a lower chlorophyll a/b ratio indicates a greater abundance of LHC. In young, but not adult, tobacco plants treated with 5 mm Cl^−^, chlorophyll a and chlorophyll b contents were significantly higher (Figures S7a–c), followed by a lower chlorophyll a/b ratio compared with SP‐ and N‐treated plants (Figure S7d). Notably, photosynthetic pigments were more abundant in Cl^−^‐treated plants during EVD and in NO_3_^−^‐treated plants during late development. This further reinforces the observation that plants during EVD are more sensitive to Cl^−^ availability compared with plants during late vegetative development, which are more sensitive to NO_3_^−^ availability. Additionally, the higher chlorophyll a + b/carotenoid ratio observed in young CL‐treated plants suggested reduced stress conditions relative to SP and N plants (Figure S7f). Overall, these findings indicate that the positive growth response to higher Cl^−^ availability during EVD underlies the enhanced protection and performance of PSII during this developmental stage.

### Chloride modifies the morphology and ultrastructure of mesophyll cells

**Figure 6:**
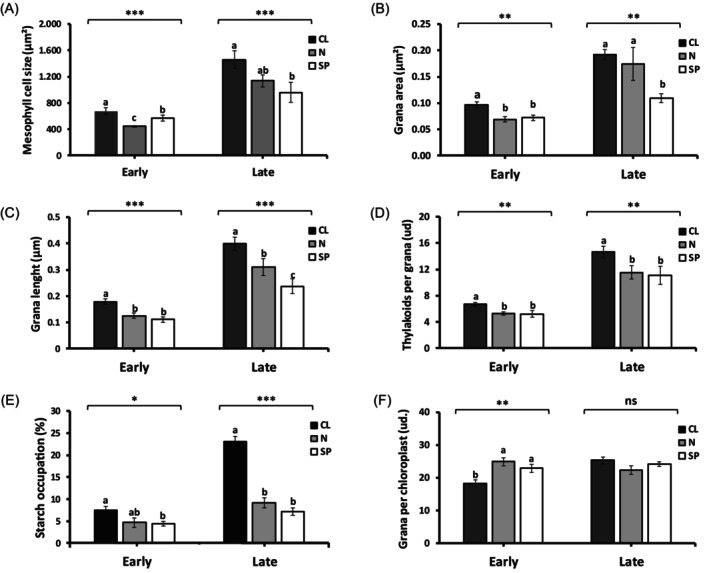
Ultrastructural alterations in chloroplasts induced by Cl^−^ during early and late vegetative development. Tobacco plants were grown with a basal nutrient solution supplemented with either 5 mm Cl^**−**^ (CL), 5 mm NO_3_^**−**^ (N), or sulphate + phosphate (SP) salts containing identical concentrations of cations. Chloroplast ultrastructure parameters were analysed from electron microscopy micrographs, including: mesophyll cell area (A); *grana* area (B); *grana* length (C); number of thylakoids per *grana* (D); starch occupation (E); and number of *grana* per chloroplast (F). Measurements were taken during early vegetative development (15 DAS) and late vegetative development (35 DAS). Data represent mean ± SE, with the number of biological and technical replicates shown in Material and Methods. Asterisks indicate statistically significant differences determined through one‐way ANOVA: ***P < 0.001, **P < 0.01, *P < 0.05, ‘ns’ (not significant) *P* > 0.05. Different letters indicate statistically significant differences between treatments (ANOVA, *P* < 0.05).

Consistent with its ability to enhance PSII performance, Cl^−^ was also found to influence plant anatomy parameters including mesophyll cell morphology and chloroplast ultrastructure. During EVD, mesophyll cell size, chloroplast *grana* size, and the number of thylakoids per *grana* were significantly lower compared with late vegetative development (Figure 6A–D). The Cl^−^ treatment promoted mesophyll cell expansion (Figure 6A), increased *grana* size (Figure 6B,C), a higher number of thylakoids *per grana* (Figure 6D), and a greater content of starch granules (Figure 6E; Figure S8a) during both early and late vegetative stages. Also, Cl^−^ reduced the number of *grana* per chloroplast during EVD but not in adult plants (Figure 6F). In adult plants, Cl^−^ also induced a higher number of smaller chloroplasts, a response not observed during EVD (Figures S8b,c; Franco‐Navarro et al., 2019). In summary, macronutrient‐level Cl^−^ nutrition enhanced photochemical activity (Figure 5, Figure S5) and biomass accumulation (Figure 1, Figures S1–S3) during EVD by promoting physiological and morphological alterations (Figures 4 and 6a, Figure S4a) and inducing ultrastructural changes in chloroplasts (Figure 6B–F), indicating that Cl^−^ facilitated the maturation of the photosynthetic machinery, improving PSII protection and performance.

## DISCUSSION

This study reveals that plant nutritional requirements vary according to the specific needs of distinct developmental stages. During EVD, plants exhibit maximal Cl^−^ demand and uptake activity, with this anion acting as a *bona fide* growth‐promoting macronutrient specifically required to boost cell expansion and PSII performance. In later stages of development, Cl^−^ demand progressively decreases, and NO_3_^−^ becomes more significant, compensating for the absence of Cl^−^ at macronutrient levels. These findings challenge conventional beliefs regarding the roles of Cl^−^ in plant nutrition and highlight its potential to enhance agricultural productivity and sustainability.

### Maximum Cl^−^ demand and optimal Cl^−^ availability enhance plant growth during EVD

Immediately following seedling establishment, plants undergo characteristic morphological and physiological changes. Morphologically, this stage is marked by higher rates of cell division and elongation, accompanied by the rapid development and expansion of the first leaves. Physiologically, plants focus on establishing a functional photosynthetic system, reflecting the transition from reliance on stored seed energy to active energy production via photosynthesis (Kitajima & Fenner, 2000). These processes are significantly enhanced by Cl^−^, as evidenced by robust growth stimulation during EVD across various herbaceous and woody plant species (Figure 1; Figures S1–S3). Although Cl^−^ is currently classified as a micronutrient, with an average content of 0.1 mg g^−1^ DW considered sufficient for adequate plant growth (Kirkby, 2023), optimal growth of a glycophyte like tobacco requires approximately 200 times more Cl^−^ during EVD (Figure 2D). This necessitates adequate Cl^−^ availability (e.g., 5 mm Cl^−^; Figure 1; Figure S1) to ensure Cl^−^ contents that are typical of a macronutrient during this developmental stage. Quantitatively, the growth‐promoting effects of Cl^−^ are strongest during EVD compared with later developmental stages (Figure 1A,D–E; Figure S1). Qualitatively, Cl^−^ deficiency during EVD cannot be compensated by NO_3_^−^ supply, in contrast to observations in later stages (Figure 1A–B; Figures S1 and S3b). Thus, Cl^−^ specifically stimulates photosynthetic parameters and biomass growth during EVD, whereas NO_3_^−^ becomes essential to promote photosynthesis and growth in later stages of development, even with low Cl^−^ availability.

In agreement with these observations, plants display a maximum Cl^−^ demand and transport activity during EVD, which decreases progressively as development advances (Figure 2). Because Cl^−^ is not metabolically assimilated, this homeostatic regulation provides two advantages. First, Cl^−^ serves as a growth‐promoting osmoticum without the risk of excessive accumulation, as strong plant growth during EVD allows the electrolyte content to be diluted (Figure 2D). Second, during later developmental stages, when relative growth rates decline, reduced Cl^−^ uptake prevents harmful accumulation (Geilfus, 2018, 2019). This regulation highlights Cl^−^ as essential to optimize plant growth during EVD, a phenomenon common across herbaceous and woody glycophytes (Figure S3; Figure 3). Chloride accumulation capacity contrasts between different species in their adult stage. Thus, leaf vegetables such as chard, spinach, and lettuce behave as strong Cl^−^ accumulators when treated with 5 mm Cl^−^, with leaf contents of 75–100 mg g^−1^ DW. Other species such as *Arabidopsis thaliana* or tomato show lower accumulations, 25 and 32 mg g^−1^ DW, respectively (Colmenero‐Flores et al., 2019). Therefore, it is interesting to note that higher Cl^−^ uptake capacity in the high‐affinity range during EVD determines similar Cl^−^ contents in these species (around 6–10 mg g^−1^ DW; Figure 3), indicating that it is a common adaptive mechanism to maximise Cl^−^ availability during EVD.

As an anion, the equilibrium potential of Cl^−^ in plant cells is quite positive, necessitating active proton‐coupled cotransport for Cl^−^ uptake under most conditions. Root Cl^−^ uptake in the low‐affinity range (e.g., at 5 mm Cl^−^) is mediated by members of the nitrate transporter 1/peptide transporter family (NPF), typically selective for NO_3_^−^ but capable of transporting Cl^−^ under low NO_3_^−^ availability (Liu et al., 2020; Xiao et al., 2021). Notably, even treatments as low as 75 μm Cl^−^ result in avid Cl^−^ accumulation during EVD (5–12 mg g^−1^ DW) in different plant species (Figure 2D; Figure 3), highlighting a strong Cl^−^ demand and the widespread activity of high‐affinity Cl^−^ uptake mechanisms in plant roots during this developmental stage. The only Cl^−^‐selective transporters involved in root uptake identified thus far belong to the NPF6 subclade, such as *Zm*NPF6.4 in maize (Wen et al., 2017) and *Mt*NPF6.5 in Medicago (Xiao et al., 2021). However, in Arabidopsis, the sole member of the NPF6 subclade, NPF6.3, is NO_3_^−^‐selective (Bouguyon et al., 2015). Interestingly, we show here strong high‐affinity Cl^−^ uptake capacity in Arabidopsis plants during EVD (Figure 3), suggesting that selective Cl^−^ transport mechanisms, distinct from those of the NPF6 subclade, are likely widespread among higher plants, and their molecular identity has yet to be established.

### Chloride drives osmotic regulation and cell expansion during EVD

Chloride‐induced growth stimulation during EVD is linked to enhanced cell expansion and PSII performance (Figures 4 and 5; Figures S4–S7). These functions are regulated in the vacuole and chloroplasts, respectively, which are the compartments where Cl^−^ shows maximum accumulation (Cakmak et al., 2023). Vigorous growth during EVD depends on high rates of cell division and expansion, which require efficient osmotic regulation and turgor. Both Cl^−^ and NO_3_^−^ are monovalent anions with similar physicochemical properties and should theoretically be equally efficient osmolytes to generate turgor at the vacuolar level. However, we verified that Cl^−^ plays a much more prominent role in promoting cell expansion, possibly due to its interaction with the phytohormone auxin (Franco‐Navarro et al., 2016). Potassium (K^+^) and Cl^−^ play critical roles in turgor maintenance and cell enlargement (Babourina et al., 1998; Franco‐Navarro et al., 2016; Fromm & Eschrich, 1988; Heslop‐Harrison & Reger, 1986; Nieves‐Cordones et al., 2019; Yamagami et al., 2004). Chloride is ubiquitous in nature, not assimilated in plant metabolism and highly mobile, which makes it a “cheap” electrolyte and therefore optimal for osmoregulation and electrical balance in plants (Franco‐Navarro et al., 2016; Wege et al., 2017; White & Broadley, 2001). Moreover, Cl^−^ enhances water retention through its unique interactions in solvation shells (Kropman & Bakker, 2001), making it an ideal osmolyte to promote cell turgor.

Cell osmoregulation is poorly compatible with anionic mineral macronutrients, such as NO_3_^−^, SO_4_^2−^, or PO_4_^3−^, which are ultimately assimilated in anabolic processes. During late development, when plants exhibit a lower RGR (as discussed below), they significantly reduce their responsiveness to Cl^−^ while markedly increasing their responsiveness to NO_3_^−^ (Figure 1B,E–F; Figure S3b). During the transition to reproductive development, the remobilization of internally stored NO_3_^−^ becomes essential to sustain amino acid and protein synthesis in developing flowers, fruits, and seeds (Masclaux‐Daubresse et al., 2008), coinciding with a peak in N demand (Masclaux‐Daubresse & Chardon, 2011). In fact, 50–90% of grain N is derived from remobilized nitrogen sources, including NO_3_^−^, during seed filling (Masclaux‐Daubresse et al., 2008). This observation supports the idea that NO_3_^−^ is incompatible with a stable osmoregulatory role in plants and explains the increased responsiveness to NO_3_^−^ during later developmental stages. Consequently, NO_3_^−^ cannot substitute for Cl^−^ in maintaining the mechanical integrity and supporting the growth of plant cells during the rapid expansion phase that characterises EVD (Figure 1A,B; Figures S1 and S3b).

Excess of NO_3_^−^ from agricultural sources is one of the main causes of aquifer pollution and eutrophication of continental and coastal waters, threatening biodiversity and the supply of drinking water (Bijay‐Singh & Craswell, 2021). In addition, NO_3_^−^ fertilisation frequently exceeds the plant growth needs, leading to over‐accumulation of NO_3_^−^ in plants, reduction of the nutritional quality of crops, and a relevant threat to human health (Essien et al., 2022; Fewtrell, 2004). Our results further reinforce the idea that the use of Cl^−^ in fertilisers can be an effective tool to significantly reduce the use of nitrates and achieve a more sustainable and healthy agriculture (Lucas et al., 2024; Neocleous et al., 2021; Rosales et al., 2020).

### Cl^−^ accelerates maturation of the photosynthetic machinery and enhances PSII performance during EVD

Chloride is the most abundant anion in the chloroplast stroma (Neuhaus & Wagner, 2000), and both the chloroplast envelope and thylakoid membrane exhibit high permeability to Cl^−^ (Bose et al., 2017; Heber & Heldt, 1981). Cl^−^ fluxes across the thylakoid membrane have been proposed to drive osmotic water movements, causing thylakoid swelling during illumination and shrinkage in darkness (Kirchhoff, 2013), having a potential role in enhancing ultrastructural control of photosynthesis (Gu et al., 2022). This can influence the conformation and structural stability of photosystem proteins, which are essential for efficient PSI electron transport, PSII repair (Kirchhoff et al., 2011), and state transitions (Chuartzman et al., 2008). Loss of function of the thylakoid Cl^−^ channel *At*CLCe in *clce* knockout mutants disrupts the organization of the thylakoid network (Herdean, Nziengui, et al., 2016). Furthermore, recent studies suggest that PSII‐OEC assembly during thylakoid development or reassembly after photodamage specifically requires Cl^−^ at levels of a macronutrient (Raven, 2020; Russell & Vinyard, 2022; Vinyard et al., 2019). The harmful, non‐regulated energy dissipation pathway Y(NO) is higher in damaged PSII due to photoinhibition (Samson et al., 2019). We observed that Cl^−^ reduces Y(NO) during both early and late vegetative development (Figure S5), pointing to a role in the protection of PSII. Other striking observations were the specific and significant stimulation of the regulated energy dissipation pathway Y(NPQ) in adult plants treated with Cl^−^ (Figure S5), a phenomenon that we are currently investigating.

Another role attributed to Cl^−^ homeostasis in the chloroplast is the regulation of the thylakoid pH gradient (ΔpH) during photosynthesis (Duan et al., 2016). Light‐induced Cl^−^ influx into the thylakoid lumen (Herdean, Nziengui, et al., 2016; Herdean, Teardo, et al., 2016) dissipates the electrical component (ΔΨ) of the proton‐motive force (PMF), thereby facilitating the buildup of the chemical H^+^ gradient (ΔpH) across the thylakoid membrane. The ΔpH is the primary component of the PMF (Wilson et al., 2021), coupling photosynthetic electron transport with ATP synthesis (Bose et al., 2017; Kramer et al., 2003). While the biochemical and biophysical effects of chloroplast Cl^−^ homeostasis have been shown *in vitro* in isolated chloroplasts or in plant mutant lines lacking functional thylakoid Cl^−^ channels, this study provides for the first time *in vivo* evidence linking Cl^−^ nutrition to plant photochemistry and growth, highlighting its critical importance during EVD.

Photosynthetic capacity is inherently lower during EVD due to incomplete development of photosynthetic structures, limited leaf area, and nutrient constraints due to reduced root development. Maturation of photosynthetic machinery according to plant age or leaf development is related to changes in the capacity for photosynthetic electron transport and phosphorylation, synthesis of chlorophylls, and Rubisco activity among others (Dickmann & Kozlowski, 1971; Pallardy, 2008; Sams & Flore, 1982; Wilson et al., 2000). During EVD, tobacco plants exhibit a third of the photosynthetic capacity observed in adult plants (Figure 4G), which was in line with reductions in key parameters such as *g*_m_ (Figure 4I), WUEi (Figure 4H), Y(II) (Figure S6a), *F*_v_/*F*_m_ (Figure 5B), and ETR (Figures 4J and 5C). Remarkably, all these parameters, together with the content of photosynthetic pigments (Figure S7), significantly improved with Cl^−^ treatment (See the same Figures), showing the electron transport capacity of PSII a significant positive response to Cl^−^ (Figure 5C) as well as a clear correlation with the increase in plant biomass during EVD (Figure 5D). In parallel, we observed structural and ultrastructural limitations in mesophyll cells during EVD, such as reduced cell size, smaller *grana*, fewer thylakoids per *grana*, and lower starch content, which were partially alleviated by Cl^−^ treatment (Figure 6A–E). This suggests that Cl^−^ contributes to alleviate mechanical and biochemical constraints in immature leaves, thereby improving PSII performance.

Higher values of the ETR/*A*_N_ ratio also point to reduced photosynthetic performance during EVD compared with adult plants (Figure S4b). Average ETR/*A*_N_ values of 6.49 and 6.39 in CL and N treatments in adult plants are lower than the most frequently observed 7.5 described by Perera‐Castro and Flexas (2023) for non‐stressed plants. An explanation for these lower ETR/*A*_N_ values is likely the high chloroplast CO_2_ concentration (*C*_c_) estimated for these treatments (data not shown), due to their higher mesophyll conductance values, and the enhanced performance of PSII, suggesting that no methodological issues were involved in the measurements.

EVD also constrains photosynthesis due to limited leaf area as well as restricted nutrient and resource availability. Consequently, Cl^−^‐induced stimulation of shoot (Figure S1a) and root organ growth (Figure S1b) creates a positive feedback loop, enhancing photosynthetic capacity and overall plant growth. During EVD, CL plants exhibited a 225 and 100% increase in leaf area (Figure S1c) compared with SP and N plants, respectively, representing a proportional improvement in light capture for photosynthesis. Additionally, CL plants showed a 150 and 83% increase in root dry weight (Figure S1b) relative to SP and N plants, respectively, enabling greater nutrient and water uptake to support their enhanced photosynthetic and growth capacity. The strong positive response of the photosynthetic capacity to nitrogen availability is a well‐known phenomenon in terrestrial plants (Hawkesford et al., 2023), which we observed in adult plants subjected to the N treatment despite the absence of Cl^−^ as a macronutrient (Figure 4G). The similar *A*_N_ in both CL and N treatments during EVD indicates that Cl^−^ is able to compensate for the lower availability of nitrogen without losing photosynthetic capacity (Figure 4G). This is also in line with a major role of turgor in the determination of growth, which is reflected in cells of larger volume (Figures 4D and 6A). Körner (2015) challenged the concept that growth was limited by photosynthesis, pointing out to turgor as the main limiting factor, as further demonstrated by other authors (Hernandez‐Santana et al., 2021; Trugman & Anderegg, 2024). This is also evident during late vegetative development, in which CL plants had significantly higher biomass (Figure 1A) and turgor (Figure 4C) than SP plants, but similar *A*_N_ (Figure 4G).

### Combined stimulation of cell expansion and PSII performance synergistically enhances EVD

Remarkably, Cl^−^‐induced stimulation of plant growth is also observed in darkness, both during seed germination (Figure S2a) and in etiolated seedlings (Figure S2b), raising the question of whether photosynthetic machinery maturation is the primary factor underlying enhanced growth during EVD. During seed germination and seedling establishment, plant growth is almost exclusively based upon cell expansion via water uptake (Bewley & Black, 1994; Cosgrove, 2005). Etiolated hypocotyl cells expand up to 100‐fold in volume, driven by vacuole enlargement rather than by new cell production (Cosgrove, 2005). We have reported that Cl^−^ is an essential electrolyte controlling osmoregulation, cell water content, turgor, and cell expansion (Franco‐Navarro et al., 2016; Colmenero‐Flores et al., 2019; Figure 4D). Supporting this, auxin‐mediated cell growth in the coleoptile of grass seedlings requires Cl^−^ uptake (Babourina et al., 1998), and anion‐channel blockers interfere with auxin responses in dark‐grown Arabidopsis hypocotyls (Thomine et al., 1997). Thus, increased Cl^−^ availability enhances growth primarily by promoting cell expansion (Figure 4D; Franco‐Navarro et al., 2016), the dominant driver of growth in germinating seeds and etiolated seedlings (Figure S2a,b). Following seedling establishment, illuminated plants during EVD (14–21 DAS) undergo characteristic morphological and physiological changes marked by higher rates of cell division, expansion of the first true leaves, and establishment of a functional photosynthetic system, reflecting the transition from reliance on stored seed energy to active energy production via photosynthesis (Kitajima & Fenner, 2000). Therefore, Cl^−^‐dependent growth stimulation in etiolated seedlings (mainly due to cell expansion) is perfectly compatible with Cl^−^‐dependent growth stimulation in illuminated plants during EVD where, besides cell expansion, other factors including the photosynthetic capacity are relevant for boosting biomass growth. Therefore, the combined promotion of photosynthetic development and cell expansion by Cl^−^ contributes additively to increased biomass during EVD.

This also raises the question of whether Cl^−^ remains equally relevant throughout the entire life cycle (not only during EVD), considering that juvenile developmental processes occur continuously in the emerging leaves of adult plants. Even when accounting for the juvenile nature of these emerging leaves, plants display their highest RGR under optimal conditions during early development—typically 0.20–0.25 g g^−1^ d^−1^ in the first 2 weeks after germination. As plants mature, their RGR declines substantially, by approximately 40–50%, when measured over the subsequent 2–4‐week period, marking a shift to slower biomass accumulation relative to total mass (Hunt & Cornelissen, 1997; Poorter & Remkes, 1990). Therefore, two beneficial effects specifically promoted by Cl^−^ combine to stimulate plant growth during EVD compared with later developmental stages. First, stimulation of higher cell expansion (Figure 4D and Figure S4a). Second, enhanced maturation of the photosynthetic apparatus during early, but not late, development (Figures 4, 5, 6 and Figures S5–S8). This is the reason why the plant exhibits a higher growth rate relative to its biomass during EVD and, therefore, a greater responsiveness to Cl^−^ (Figure 1E–F).

## CONCLUSIONS

During EVD, plants exhibit a maximal demand for Cl^−^, resulting in the highest net root uptake rate, optimally supporting cell expansion, maturation of the photosynthetic machinery, PSII performance, and biomass accumulation. Thus, Cl^−^ functions as a *bona fide* essential macronutrient during EVD, a period when its deficiency cannot be mitigated by additional NO_3_^−^ supply. As plants transition to late vegetative and reproductive stages, Cl^−^ demand gradually declines, likely minimizing the risk of excessive accumulation while allowing NO_3_^−^ supplementation to compensate for any deficiency. This novel understanding of the distinct roles of Cl^−^ and NO_3_^−^ across developmental stages has significant implications for optimizing agronomic practices, particularly by reducing dependence on nitrogen fertilisers. These findings pave the way for further research into nutrient management strategies and challenge preconceived notions about the significance of Cl^−^ in plant nutrition.

## MATERIAL AND METHODS

### Plant material and growth conditions

Seed germination in pots and growth conditions of tobacco (*Nicotiana tabacum* var. light Habana), arabidopsis (*Arabidopsis thaliana* Col‐0 ecotype), tomato (*Solanum lycopersicum L*. var. Ailsa craig), lettuce (*Lactuca sativa* ssp. *longifolia* Lam.) and those plants listed in Table S1 were as described in Franco‐Navarro et al. (2016). Plants were grown under greenhouse conditions with regular temperature of 24 ± 3°C/17 ± 2°C (day/night), relative humidity 60 ± 10% (EL 1‐USB Data logger Lascar Electronics Inc., Erie, PA, USA, https://www.lascarelectronics.com/), a 14/10 h photoperiod with a photosynthetic photon flux density [average photosynthetically active radiation (PAR)] of 300–350 μmol m^−2^ s^−1^ (quantum sensor, LI‐6400; Li‐COR, Lincoln, NE, USA, https://www.licor.com/), and a luminous emittance of 9000–10 000 lx (Digital Lux Meter, LX1010B; Carson Electronics, Valemount, BC, Canada, http://www.carsons.ca/).

Most experimental approaches were performed with tobacco plants, establishing 13–21 DAS as EVD and 35 as late vegetative development. Tobacco plants were also grown up to the reproductive stage (60–67 DAS) when required. In arabidopsis plants, we established 15 DAS as EVD and 30 DAS as late vegetative development. In other plant species, harvesting times at different developmental stages were as indicated in Table S1. The RGR was calculated in tobacco plants within the 9–27 DAS interval for EVD, and between the 27–64 DAS interval for late development.

The nutritional treatments were as previously described (Franco‐Navarro et al., 2016). In brief, a basal nutrient solution (BS), containing all plant nutrients, including Cl^−^ to fulfill essential micronutrient requirements (75 μm; Johnson et al., 1957), was alternatively supplemented with either Cl^−^ salts (5 mm Cl^−^, CL treatment), NO_3_^−^ salts (5 mm NO_3_^−^, N treatment), or a mix of sulphate (SO_4_^2−^) + phosphate (PO_4_^3−^) salts (1.25 mm SO_4_^2−^ + 1.25 mm PO_4_^3−^; SP treatment). The three treatments, CL, N, and SP, contained the same balance of cations (see the complete content of mineral nutrients in Table S2a). For the Cl^−^ gradient treatment, SO_4_^2−^ + PO_4_^3−^ salts were used to replace Cl^−^ salts to maintain identical concentration of cations (Table S2b). For evaluation of the seed germination rate of tobacco and tomato plants, a BS containing 10 mm NO_3_^−^ was supplemented with either 5 mm Cl^−^ salts (CL) or the corresponding SP control treatment.

Woody plants were germinated and grown under *in vitro* conditions. Citrus seeds of the rootstock citrange Carrizo were surface‐sterilized with 4% NaOCl and 0.1% tween for 10 min, and three subsequent washes with distilled water were applied. Seeds were sown in sterile glass tubes containing SP, N, and CL solutions supplemented with 6 g/L plant agar. After 2 weeks vernalization, glass tubes were transferred to a room chamber and incubated for 45 days to evaluate *in vitro* germination and growth. Olive seeds were surface‐sterilized and germinated *in vitro* according to the methodology described by Díaz‐Rueda et al. (2020).

### Net uptake rate of Cl^−^ and other mineral nutrients

Seeds of wild‐type *Arabidopsis thaliana* (Col‐0) plants were surface‐sterilized and sown on vertical agar plates consisting of SP medium supplemented with agar 0.8% and sucrose 1%. Agar plates were incubated for 10–14 days in a growth chamber (24°C, 40–60% relative humidity, 16 h/8 h photoperiod with a photosynthetic photon flux density of 150–200 μmol m^−2^ s^−1^) prior to being transferred to hydroponics conditions, in which plants were acclimatized before carrying out experimental treatments. For the determination of the net uptake rate of Cl^−^, NO_3_^−^, and K^+^ during EVD (Figure 2A), 10 DAS seedlings grown in agar plates were transferred to 24‐well cell culture microplates (Grenier bio‐one) containing modified SP or CL solutions (Table S3), and plants were left to grow for 5 additional days before starting determinations in 15 DAS plants. Plants were transferred to fresh SP and CL solutions and incubated for 24 and 96 h. Then, plants were removed to calculate the volume of medium consumed by each plant. This volume was supplemented with ultrapure Milli‐Q water before proceeding to the quantification of Cl^−^, NO_3_^−^, and K^+^ concentrations in the nutrient solution to determine the absolute amount of ions taken up by the plant. Net uptake rates were calculated based on the amount of each nutrient consumed relative to the root biomass and the elapsed time. For determinations during late vegetative development, 12 DAS seedlings were transferred from agar plates to hydroponics jars containing SP or CL solution, and plants were left to grow for an additional 9 days. Subsequently, plants were placed individually into 50 ml Falcon‐type tubes and left to grow for four additional days before starting determinations in 25 DAS plants, as explained before.

For short‐term Cl^−^ treatments and plant Cl^−^ content determination (Figure 2B), plants were transferred to hydroponic conditions in SP medium and were left to grow for an additional 6, 16, or 28 days to respectively obtain plants in different developmental stages: 20 DAS (early vegetative); 30 DAS (late vegetative); 42 DAS (reproductive). Plants were transferred to CL medium for 12 h and harvested for quantification of total Cl^−^ content.

### Extraction of xylem sap from tobacco plants

Xylem sap sampling was performed at 15, 25, 36, 60, and 75 DAS. Tobacco plants grown in pots with solid substrate as described in Franco‐Navarro et al. (2016) were partially submerged in the appropriate nutrient solutions (Table S4) within a closed container in order to generate an atmosphere saturated with 100% humidity. Three hours after transferring the pots to the respective containers, xylem sap secretion was induced by sectioning the aerial part of the plant with a scalpel at the base of the stem as previously described (Cubero‐Font et al., 2016). Xylem sap was further collected at 0.5, 1, 2, 4, 6, and 12 h after stem sectioning and diluted three times in ultrapure water prior to performing Cl^−^ and NO_3_^−^ content determinations.

### Nutrient content quantification

Plant samples were dried in a forced‐air oven at 75°C to obtain the dry weight (DW) and kept dry until use. Along with xylem sap samples, Cl^−^ and NO_3_^−^ content was obtained spectrophotometrically (OMEGA) as previously reported (Franco‐Navarro et al., 2016) based on the procedures described by Cataldo et al. (1975) and Frankenberger et al. (1996). Units were expressed in millimolar or mg per gram of DW.

### Photosynthesis, leaf water and gas exchange, chlorophyll fluorescence and content

Leaf osmotic potential (Ψ_π_) was acquired from the leaf sap obtained from leaf discs of six individuals per treatment. Leaf sap was extracted from leaf discs by transferring the samples, placed in 0.5 ml microcentrifuge tubes, from a block heated to 90°C to liquid nitrogen. Tube caps were sealed with parafilm to avoid water evaporation. This thermal shock was repeated five times, and leaf sap was collected in 1.5 ml tubes by centrifugation and filtration of tissue debris. For leaf water potential (Ψ_w_) measurements, leaves were bagged with a sealed plastic bag during 20–30 min before collection (Begg & Turner, 1970). Samples were all double‐bagged in a plastic bag saturated with water vapor and carried to the laboratory in an insulated box. Either Ψ_π_ or Ψ_w_ was recorded using the dew‐point microvoltimeter (model HR‐33 T, Wescor, UT, USA) and the C‐52 sample chamber as previously described in Colmenero‐Flores et al. (1999). Leaf turgor (or pressure) potential (Ψ_p_) was determined from the Ψ_w_ and Ψ_π_ values according to the Equation:

$$\Psi_{p}\;\left(\mathrm{MPa}\right)=|\Psi_{\pi }|-|\Psi_{w}|$$

Leaf gas exchange parameters were determined on fully expanded leaves from *N. tabacum* plants of 13 and 35 DAS (*n* = 8) between 11:00 h and 14:00 h using the open gas exchange system (Li‐Cor, Lincoln, NE, USA) equipped with a fluorescence chamber (LI‐6400‐40, Li‐Cor, inc). Photosynthesis was induced with saturating light at a photosynthetic photon flux density (PPFD) of 1000 μmol m^−2^ s^−1^ (10% blue light) with 550 μmol mol^−1^ CO_2_ surrounding the leaf (*C*_a_) and the flow rate was set at 300 μmol s^−1^. For a correct data measurement, quantifications required between 2 and 5 min for gas exchange to reach steady state conditions. All the independent experiments were subjected to the same conditions and were constant for net photosynthetic rate (*A*_N_; μmol CO_2_ m^−2^ s^−1^) and stomatal conductance (*g*_s_; mol H_2_O m^−2^ s^−1^). Subsequently, the ratio between *A*_N_ and *g*_s_ was calculated to obtain the intrinsic water‐use efficiency value (WUEi; μmol CO_2_ mol^−1^ H_2_O). Mesophyll diffusion conductance to CO_2_ (*g*_*m*_) was calculated by simultaneously measuring leaf gas exchange and chlorophyll fluorescence, as described by Harley et al. (1992) with modifications. This method required an open gas exchange system with an integrated fluorescence chamber head (LI‐6400‐40, LI‐COR, Lincoln, NE, USA; https://www.licor.com/).

To determine Photosystem II functionality, photochemical [Y(II)] and non‐photochemical [Y(NPQ) and Y(NO)] parameters were measured in tobacco and Arabidopsis plants at room temperature after dark incubation for 15 min by a pulse amplitude modulation fluorometer (Imaging‐PAM M series, Walz, Effeltrich, Germany) as described by Kramer et al. (2004). The *F*_v_/*F*_m_ ratio or maximum potential quantum efficiency of PSII photochemistry was calculated using a pulse of blue light (450 nm) at 10 000 μE m^−2^ s^−1^ light intensity for 0.6 s.

For determination of chlorophyll content in tobacco plants, frozen leaf samples were ground in a chilled mortar with 80% acetone and subsequently centrifuged at 13000 *g* for 10 min. The content of chlorophylls and carotenoids obtained in each supernatant was measured as indicated in Lichtenthaler and Buschmann (2001) and referred to as mg of pigment per g of DW.

### Microscopy and mesophyll cell anatomy

To evaluate anatomical changes in epidermal cells of tobacco leaves, epidermal impressions from the abaxial surface were carried out to determine the size and density of stomatal and epidermal cells (*n* = 65) through optical microscopy micrographs (Leica DM 2000, Wetzlar, Hesse, Germany) according to procedures described in Franco‐Navarro et al. (2016). For transmission electron microscopy analysis (TEM), samples of tobacco leaves (1 × 2 mm) were fixed with 2% glutaraldehyde in 0.1 M cacodylate buffer, pH 7.3 for 5 h, washed in the same buffer, and kept overnight at 4°C. Postfixation consisted of 0.1 M cacodylate buffer, pH 7.3, with osmium tetroxide 1%. Fixed material was dehydrated by increasing acetone series and embedded in an epoxy embedding medium kit (Sigma‐Aldrich, St. Louis, MO, USA). Sections were obtained with an ultramicrotome (Reichert‐Jung Ultracut, Wien, Austria) with a diamond blade. The grids were contrasted with 2% aqueous uranyl acetate for 8 min and analysed with an electron microscope (Zeiss Libra 120, Jena, Germany) at 80 kV (CITIUS, University of Seville).

TEM micrographs (250×) were used to measure the size of spongy mesophyll cells and the number of chloroplasts per cell (*n* = 15–25). TEM micrographs (2500×) were used for the measurement of chloroplast ultrastructure parameters, including the *grana* area and length (*n* = 225–250), the number of thylakoids per *grana* and *grana* per chloroplast (*n* = 30–35), as well as the size of starch granules (*n* = 15–25). All the micrographs obtained for the study of tobacco leaf anatomy were analysed with the ImageJ image processing software developed at the National Institutes of Health and the Laboratory for Optical and Computational Instrumentation (LOCI, University of Wisconsin).

### Statistical analyses

Datasets were analysed using STATGRAPHICS Centurion XVI software (StatPoint Technologies, Warrenton, VA, USA). Results were denoted non‐significant (ns) when *P* ≥ 0.05, and significance levels were indicated as follows: *** *P* ≤ 0.001; ** *P* ≤ 0.01; *P* ≤ 0.05; ‘ns’ (not significant) *P* > 0.05. The Shapiro–Wilk (*W*) test was used to verify the normality of the results, and the Levene test to determine the homogeneity of variance. Data were transformed when homogeneity of variance or normality was not achieved using the functions 1/x, √x, and ln(x). One‐way analysis of variance (ANOVA) or multivariate ANOVA (MANOVA) was used considering DAS and treatments as grouping factors. Means were compared by Tukey's HSD *post hoc* test. When homogeneity of variance or normality was not reached after data transformation, non‐parametric Kruskal–Wallis ANOVA was performed by *post hoc* Mann–Whitney (*U* test). For the linear regression performed to analyse the relationship between ETR‐II (dependent variable) and total biomass (independent variable), the statistical significance of the regression coefficient was assessed using the Student's *t* test, and the model fit was evaluated using the coefficient of determination (*R* ^2^). Results represent the mean of at least three plants per treatment, and it was reproducible at least in two independent experiments.

## AUTHOR CONTRIBUTIONS

PP‐T, ML, JDF‐N, DR‐J, FJ M‐R., and PD‐R performed the experiments and participated in the conception of experiments and research plans. Supervision of the experiments was conducted by JMC‐F., RA, and AD‐E. PP‐T, JMC‐F, and RA wrote the first draft of the article, which was further revised by JDF‐N., ML, AD‐E., MAR, and JMC‐F. The research line was conceived by JMC‐F. The funds to finance the project were obtained by JMC‐F.

## Supporting information


**Figure S1.** Macronutrient Cl^−^ nutrition is essential for optimal plant growth during early vegetative development, whereas NO_3_^−^ nutrition becomes more relevant in adult plants.
**Figure S2.** Effect of macronutrient Cl^−^ nutrition during seed germination and the growth of etiolated seedlings.
**Figure S3.** Effect of macronutrient Cl^−^ nutrition on the growth of various herbaceous and woody species during early development.
**Figure S4.** Other physiological and morphological changes driven by Cl^−^ nutrition during early and late vegetative development.
**Figure S5.** Regulation of photochemical and non‐photochemical parameters by different nutritional treatments and development.
**Figure S6.** Regulation of the PSII performance: comparison of early versus late vegetative development.
**Figure S7.** Content of pigments in tobacco leaves.
**Figure S8.** Changes in leaf anatomy and chloroplast ultrastructure driven by Cl^−^ nutrition during early and late vegetative development.
**Table S1.** Relation of plant species used in 3.
**Table S2.** (a) Content of mineral nutrients in the CL, N, and SP treatments. (b) Nutrients composition in solutions used for the Cl^−^ gradient treatments.
**Table S3.** Composition of modified SP and CL treatments for net uptake rate experiments in *Arabidopsis thaliana* plants.
**Table S4.** Relation of nutritional treatments for quantification of ion content in xylem sap secretion extracts in tobacco plants.

## Data Availability

Data sharing not applicable to this article as no datasets were generated or analysed during the current study.
