# Supplementary material for: Plants maximise chloride uptake during early vegetative development to stimulate cell expansion, maturation of the photosynthetic apparatus, and growth
Source: Plant J. 2025 Aug 30;123(5):e70378. doi: 10.1111/tpj.70378 (PMC12397901; doi:10.1111/tpj.70378)
Supplement: Supplementary file 1 — Figure S1. Macronutrient Cl− nutrition is essential for optimal plant growth during early vegetative development, whereas NO3 − nutrition becomes more relevant in adult plants. Figure S2. Effect of macronutrient Cl− nutrition during seed germination and the growth of etiolated seedlings. Figure S3. Effect of macronutrient Cl− nutrition on the growth of various herbaceous and woody species during early development. Figure S4. Other physiological and morphological changes driven by Cl− nutrition during early and late vegetative development. Figure S5. Regulation of photochemical and non‐photochemical parameters by different nutritional treatments and development. Figure S6. Regulation of the PSII performance: comparison of early versus late vegetative development. Figure S7. Content of pigments in tobacco leaves. Figure S8. Changes in leaf anatomy and chloroplast ultrastructure driven by Cl− nutrition during early and late vegetative development. Table S1. Relation of plant species used in 3. Table S2. (a) Content of mineral nutrients in the CL, N, and SP treatments. (b) Nutrients composition in solutions used for the Cl− gradient treatments. Table S3. Composition of modified SP and CL treatments for net uptake rate experiments in Arabidopsis thaliana plants. Table S4. Relation of nutritional treatments for quantification of ion content in xylem sap secretion extracts in tobacco plants. [file TPJ-123-0-s001.pdf]

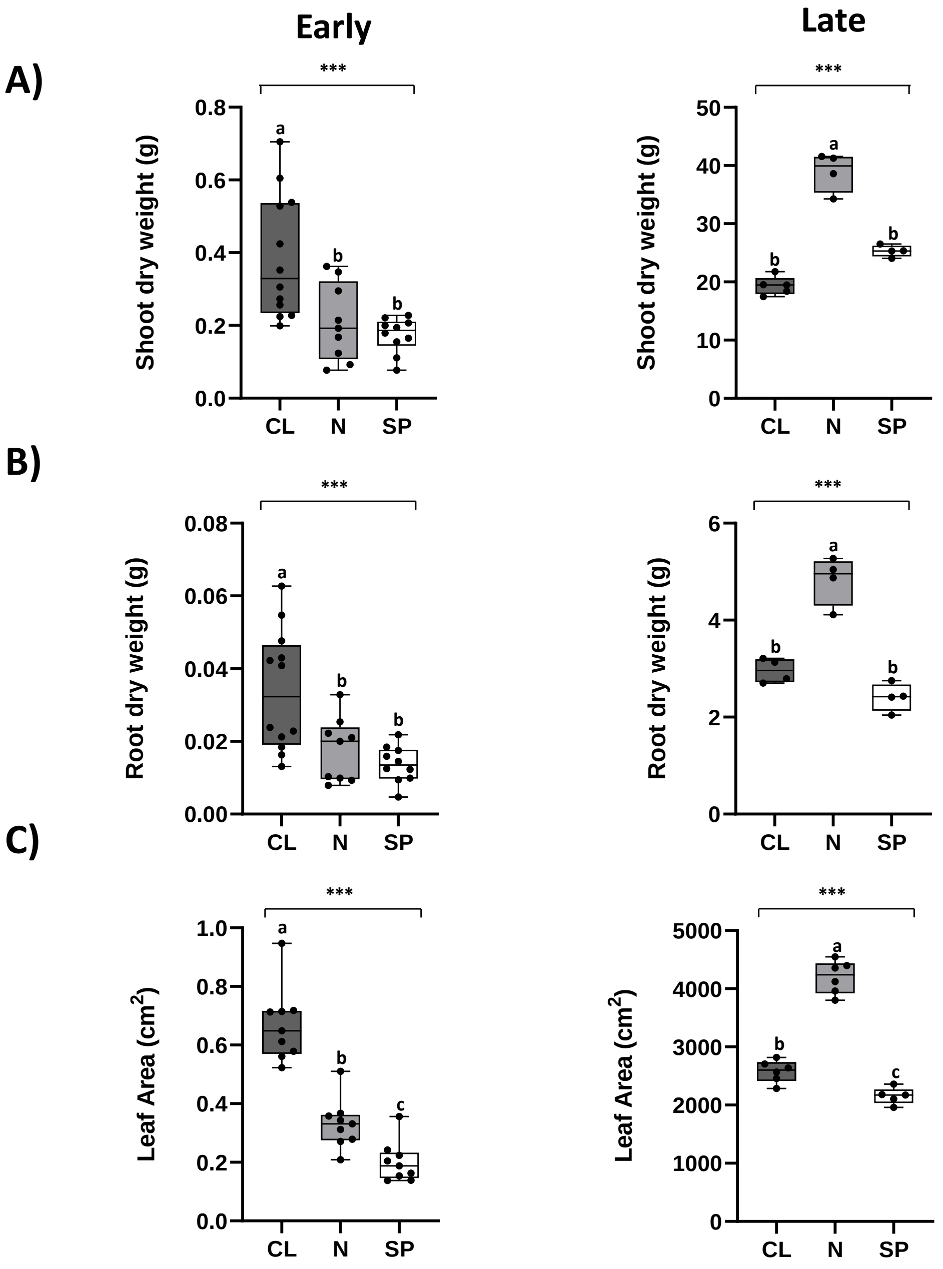

Figure S1. Macronutrient Cl^{-} nutrition is essential for optimal plant growth during early vegetative development, whereas NO_3^{-} nutrition becomes more relevant in adult plants. *Nicotiana tabacum* plants were treated with a basal nutrient solution supplemented with either 5 mM Cl^{-} (CL), 5 mM NO_3^{-} (N), or sulphate + phosphate (SP) salts containing identical concentrations of cations. Dry weight of the plant shoot **(A)**, the plant root **(B)** and leaf area **(C)** were measured during early vegetative development (15 DAS, Days After Sowing) and late development (65 DAS). Data are presented as mean ± SE (n = 3 - 9). Asterisks indicate statistically significant differences (one-way ANOVA, ***P < 0.001). Different letters indicate statistically significant differences between treatments (ANOVA, P < 0.05).

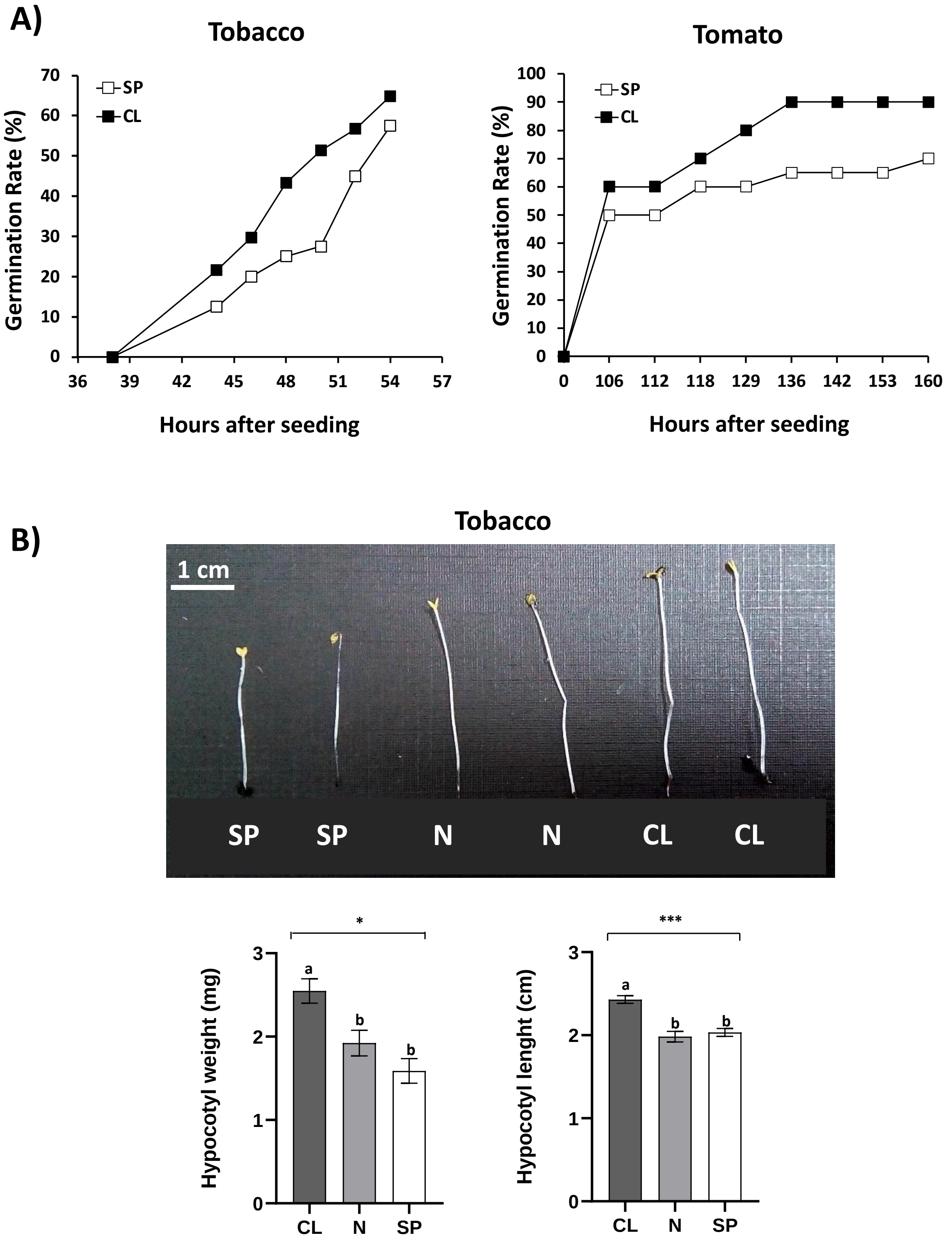

Figure S2. Effect of macronutrient Cl^{-} nutrition during seed germination and the growth of etiolated seedlings. **(A)** Germination rate of tobacco and tomato seeds exposed to a basal nutrient solution containing 10 mM NO_3^{-} supplemented with either 5 mM Cl^{-} (CL) or sulphate + phosphate (SP) salts with identical concentrations of cations. **(B)** Fresh weight and hypocotyl length of tobacco seedlings grown in darkness and treated with a basal nutrient solution supplemented with either 5 mM Cl^{-} (CL), 5 mM NO_3^{-} (N), or sulphate + phosphate (SP) salts containing identical concentration of cations. Data are presented as mean ± SE (n = 80 - 90). Asterisks indicate statistically significant differences (one-way ANOVA, ***P < 0.001; *P < 0.05). Different letters indicate statistically significant differences between treatments (ANOVA, P < 0.05).

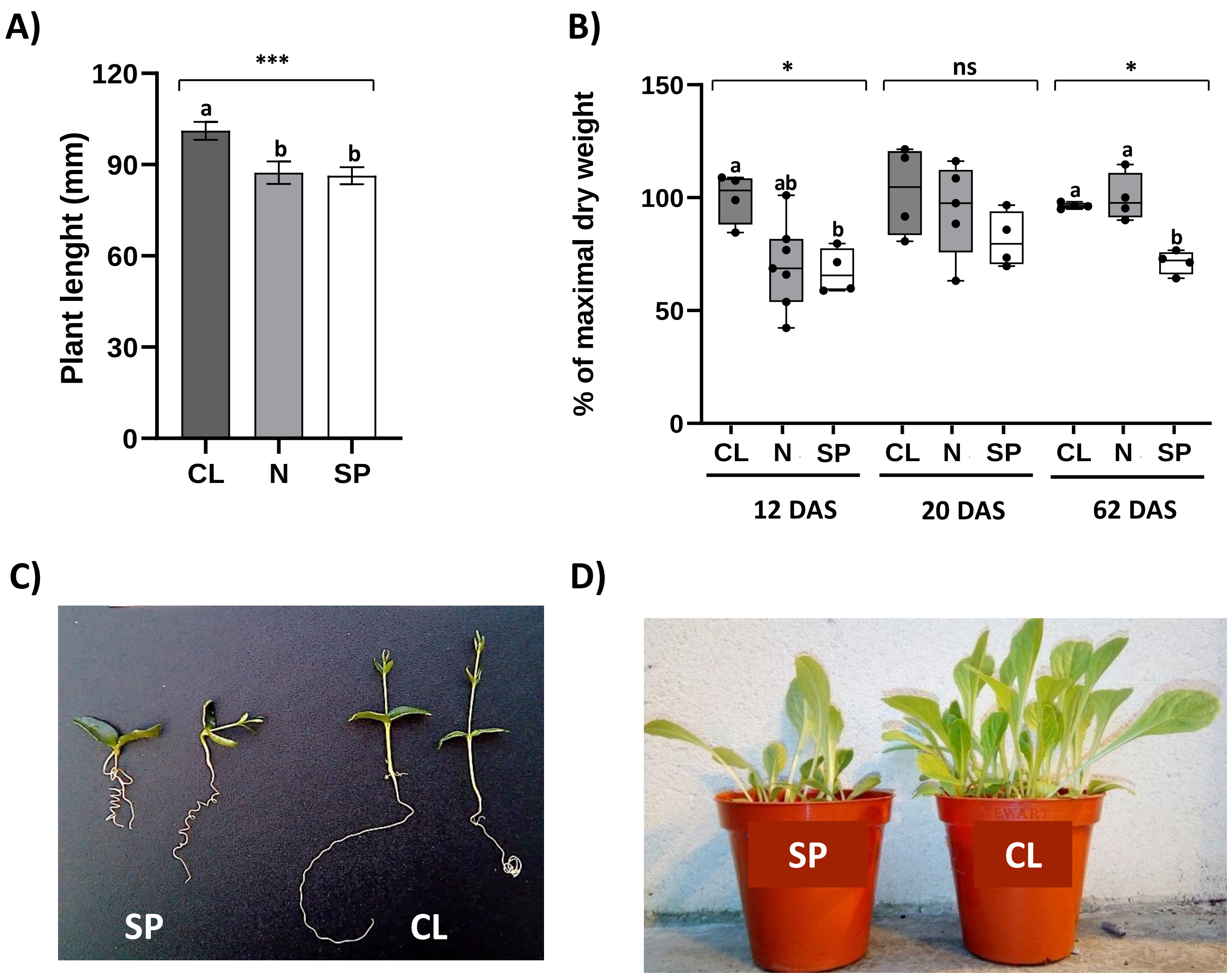

Figure S3. Effect of macronutrient Cl^{-} nutrition on the growth of various herbaceous and woody species during early development. Plants were treated with a basal nutrient solution supplemented with either 5 mM Cl^{-} (CL), 5 mM NO_3^{-} (N), or sulphate + phosphate (SP) salts containing identical concentrations of cations. **(A)** Length of *in vitro*–grown seedlings of the citrus rootstock Carrizo citrange at 60 DAS (Days After Sowing). **(B)** Relative growth (% of maximal dry biomass) of tomato plants measured at different developmental stages. **(C)** *In vitro*–grown olive seedlings at 45 DAS. **(D)** Lettuce plants at 23 DAS. Asterisks indicate statistically significant differences (one-way ANOVA, ***$P < 0.001$; *$P < 0.05$; 'ns' (not significant) $P > 0.05$). Different letters indicate statistically significant differences between treatments (ANOVA, $P < 0.05$; mean ± SE, $n = 4$-6).

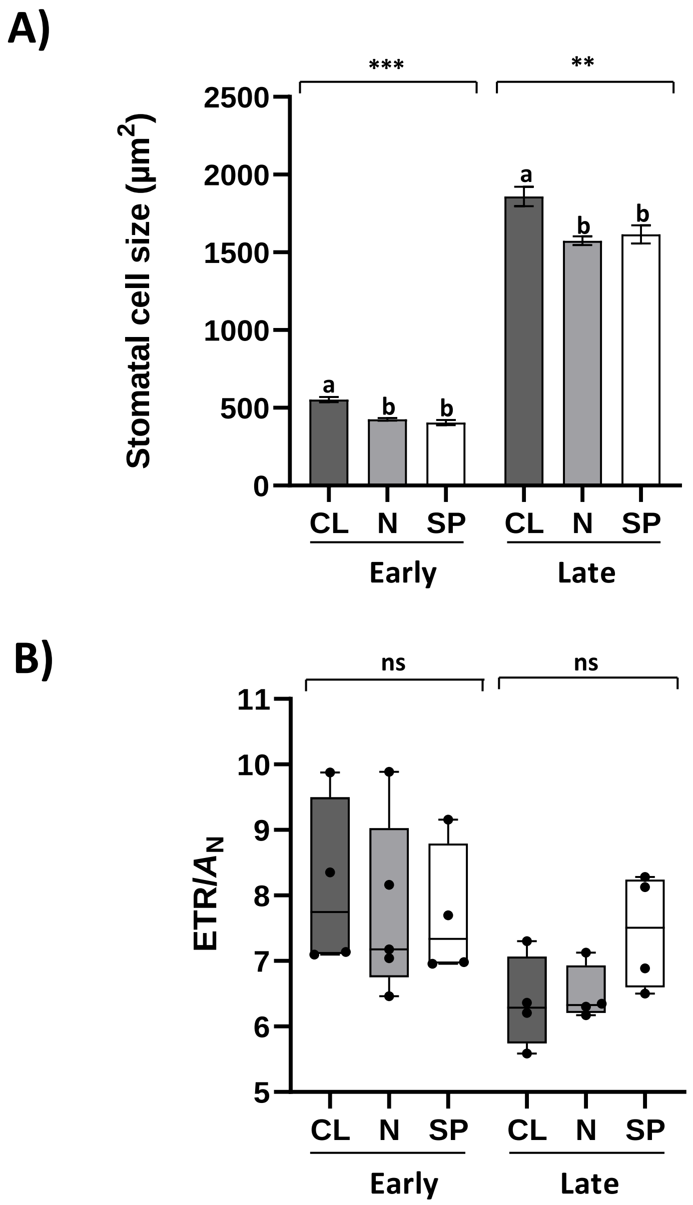

Figure S4. **Other physiological and morphological changes driven by Cl^{-} nutrition during early and late vegetative development**. Tobacco plants were grown with a basal nutrient solution supplemented with either 5 mM Cl^{-} (CL), 5 mM NO_3^{-} (N), or sulphate + phosphate (SP) salts containing identical concentrations of cations. **(A)** Morphological changes in stomatal cells were characterised during early vegetative development (15 DAS, Days After Sowing) and late vegetative development (35 DAS; *n* = 25 – 35). **(B)** ETR/A_N (*n* = 4). Asterisks indicate statistically significant differences determined through one-way ANOVA (***$P < 0.001$, **$P < 0.01$, 'ns' (not significant) $P > 0.05$; mean ± SE). Different letters denote statistically significant differences between treatments (ANOVA, $P < 0.05$).

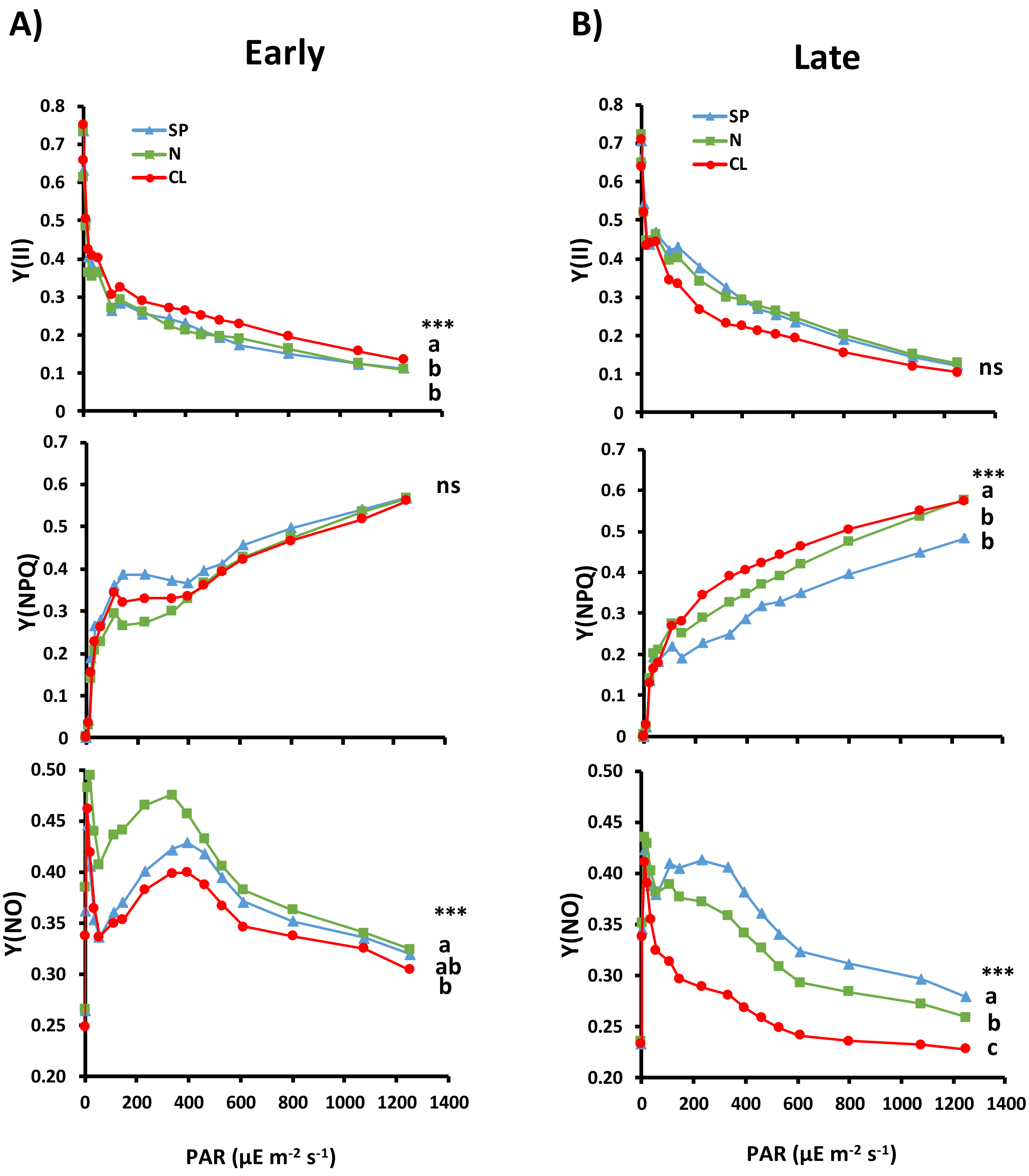

Figure S5. **Regulation of photochemical and non-photochemical parameters by different nutritional treatments and development.** Tobacco plants were grown with a basal nutrient solution supplemented with either 5 mM Cl^-, 5 mM NO_3^-, and sulphate + phosphate salts containing identical concentrations of cations. The relative contribution of the effective quantum yield of PSII [Y(II)], the non-photochemical quenching or yield of regulated energy dissipation of PSII [Y(NPQ)], and the yield of non-regulated energy dissipation of PSII [Y(NO)] were measured in plants illuminated with different light intensities. Plants were grown under 230 PAR illumination before fluorometric measurements were recorded with the image PAM device under varying light intensities. Different letters indicate statistically significant differences (MANOVA,****P* < 0.001, 'ns' (not significant) *P* > 0.05; mean ± SE, *n* = 4-6).

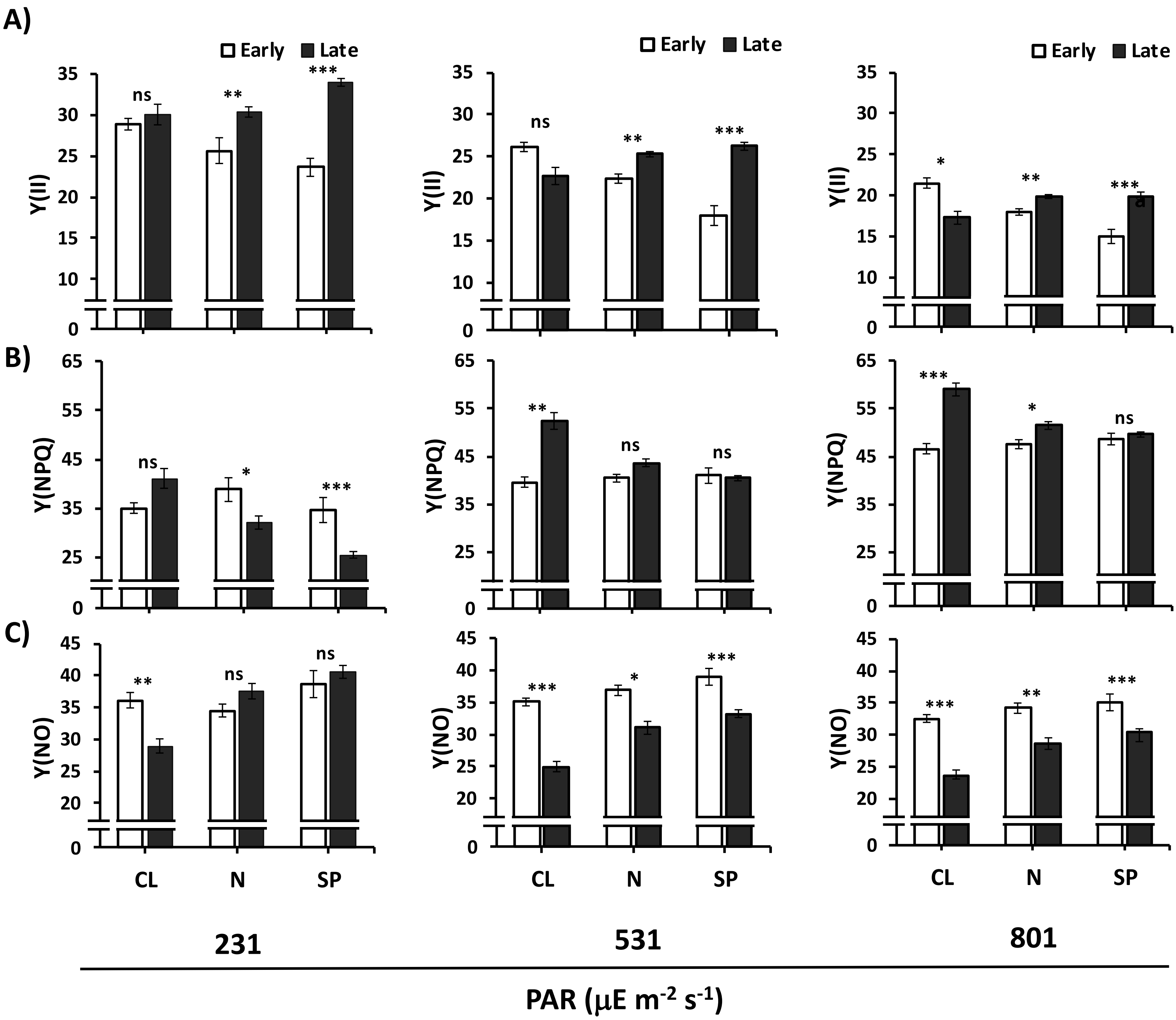

Figure S6. **Regulation of the PSII performance: comparison of early *vs* late vegetative development.** Tobacco plants were grown with a basal nutrient solution supplemented with either 5 mM Cl^- (CL), 5 mM NO_3^- (N) and sulphate + phosphate (SP) salts containing identical concentrations of cations. The relative contributions of the effective quantum yield of PSII [Y(II)] **(A)**; the non-photochemical quenching or yield of regulated energy dissipation of PSII [Y(NPQ)] **(B)**; and the yield of non-regulated energy dissipation of PSII [Y(NO)] **(C)** were evaluated. Plants were grown under 230 PAR illumination before fluorometric measurements were recorded using an image PAM device at varying light intensities: 231, 531, and 801 PAR (μE m^{-2} s^{-1}). Asterisks indicate statistically significant differences determined through one-way ANOVA, ***$P < 0.001$, **$P < 0.01$, *$P < 0.05$, 'ns' (not significant) $P > 0.05$; mean ± SE, $n = 4$.

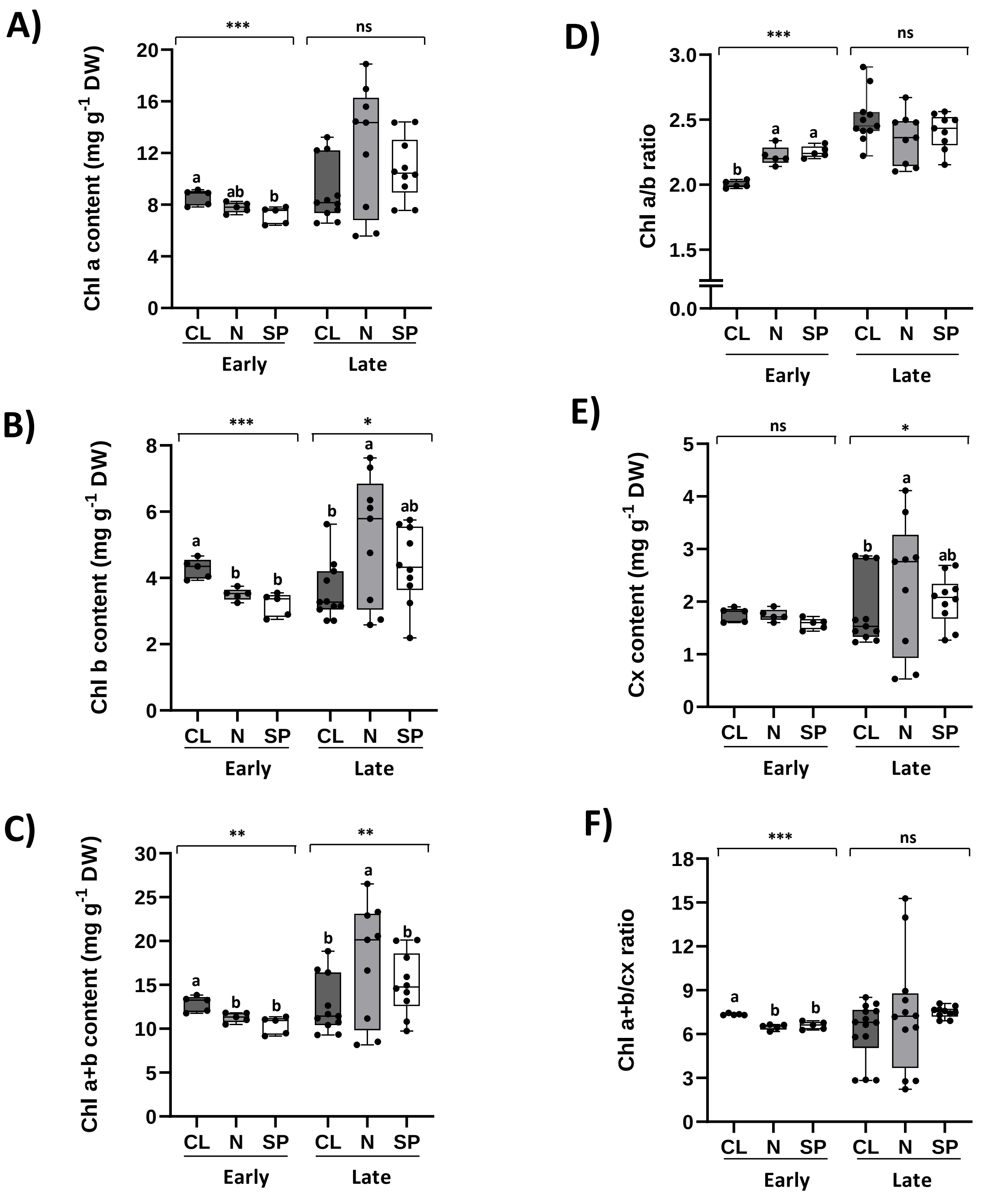

Figure S7. Content of pigments in tobacco leaves. Tobacco plants were grown with a basal nutrient solution supplemented with either 5 mM Cl^- (CL), 5 mM NO_3^- (N) or sulphate + phosphate (SP) salts containing identical concentrations of cations. The following pigment contents and ratios were measured: chlorophyll *a* **(A)**; chlorophyll *b* **(B)**; total chlorophylls **(C)**; chlorophyll a:b ratio **(D)**; carotenoids **(E)**; total chlorophylls:carotenoids ratio **(F)**. Plants were grown under 300 PAR illumination for 13 days (early development) or 35 days (late development) before harvesting. Data represent mean values ± SE (*n* = 5 - 15). Asterisks indicate statistically significant differences determined through one-way ANOVA: ***P < 0.001, **P < 0.01, *P < 0.05, ‘ns’ (not significant) P > 0.05; mean ± SE, *n* = 4. Different letters indicate statistically significant differences between treatments (ANOVA test, P < 0.05).

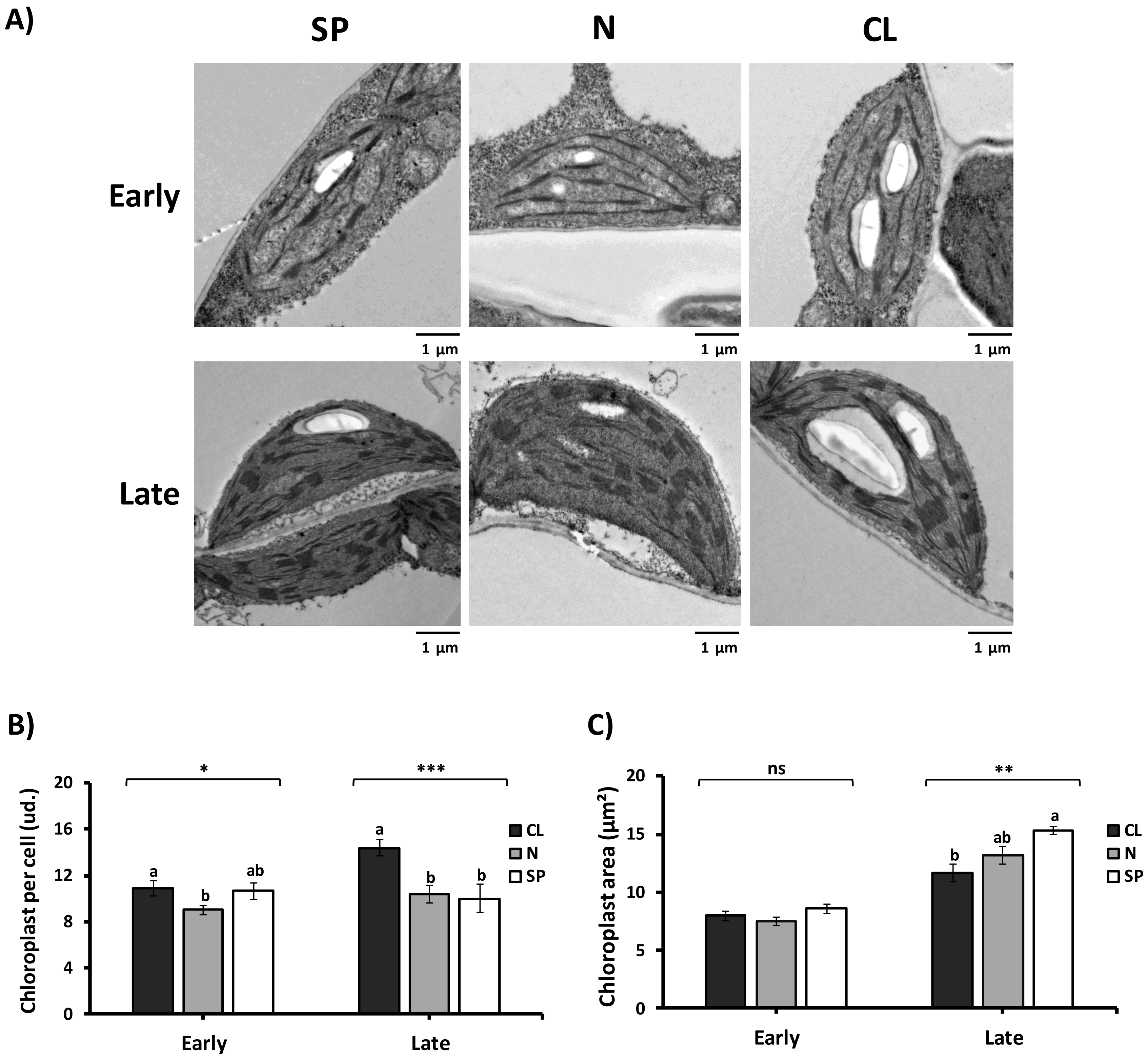

Figure S8. Changes in leaf anatomy and chloroplast ultrastructure driven by Cl^- nutrition during early and late vegetative development. Tobacco plants were grown with a basal nutrient solution supplemented with either 5 mM Cl^- (CL), 5 mM NO_3^- (N), and sulphate + phosphate (SP) salts containing identical concentrations of cations. Data were recorded during early (13 DAS, Days After Sowing) and late vegetative development (35 DAS). Chloroplast ultrastructure was analysed from electron microscopy micrographs. **(A)** General aspect of chloroplasts according to the plant developmental stage or the nutritional treatment; **(B)** number of chloroplasts per cell; **(C)** chloroplast surface area. Asterisks indicate statistically significant differences determined through one-way ANOVA (***$P < 0.001$, **$P < 0.01$, *$P < 0.05$, 'ns' (not significant) $P > 0.05$; mean ± SE, n = 25 - 35). Different letters indicate statistically significant differences between treatments (ANOVA test, $P < 0.05$).

Table S1. Relation of plant species used in Fig.3.

			Harvest (DAS)	
**Group**	**Family**	**Specie**	**Early**	**Late**
Dicotyledoneae	Apiaceae	*Apium graveolens*	19	62
Dicotyledoneae	Asteraceae	*Lactuca sativa*	10	64
Dicotyledoneae	Brassicaceae	*Arabidopsis thaliana* var. columbia	10	64
Dicotyledoneae	Chenopodiaceae	*Beta vulgaris* var. cicla	9	64
Dicotyledoneae	Chenopodiaceae	*Spinacia oleracea*	9	64
Dicotyledoneae	Cucurbitaceae	*Cucurbita pepo*	8	62
Dicotyledoneae	Solanaceae	*Nicotiana tabacum* var. light habana	14	64
Dicotyledoneae	Solanaceae	*Nicotiana tabacum* var. xanthi	14	64
Dicotyledoneae	Solanaceae	*Solanum lycopersicum* var. campbell33	13	62
Dicotyledoneae	Solanaceae	*Capsicum frutescens*	12	62
Dicotyledoneae	Solanaceae	*Solanum melongena*	18	62
Monocotyledoneae	Poaceae	*Lolium perenne*	10	64

DAS, Days After Sowing.

Table S2A. Content of mineral nutrients in the CL, N and SP treatments.

Nutrients (mM)	SP	N	CL
Na^{+}	0.1	0.1	0.1
K^{+}	4.49	4.49	4.49
NO_3^{-}	5.25	10.25	5.25
PO_4^{-}	1.93	0.68	0.68
SO_4^{-}	3.01	1.14	1.14
Ca^{2+}	2.62	2.62	2.62
Mg^{2+}	2.62	1.62	1.62
Cl^{-}	0.075	0.075	5.0

Table S2B. Nutrient composition in solutions used for the Cl^{-} gradient treatments.

Nutrients (mM)	0.075	0.1	0.5	1.0	2.5	5	10
Na^{+}	0.1	0.1	0.1	0.1	0.1	0.1	0.1
K^{+}	4.486	4.486	4.486	4.486	4.486	4.486	4.486
Ca^{2+}	2.625	2.625	2.625	2.625	2.625	2.625	3.25
Mg^{2+}	2.625	2.625	2.625	2.625	2.625	2.625	3.25
Cl^{-}	0.075	0.1	0.5	1.0	2.5	5.0	10.0
NO_3^{-}	5.250	5.250	5.250	5.250	5.250	5.250	5.250
SO_4^{2-}	3.014	2.976	2.737	2.639	2.076	1.139	1.139
PO_4^{3-}	1.928	1.903	1.803	1.678	1.303	0.678	0.678
S+P	4.932	4.879	5.450	4.317	3.379	1.817	1.817

The reduction of Cl^{-} in the solutions was done by replacing it with PO_4^{-} and SO_4^{-} salts in such a way that the same concentration of cations was always maintained in all treatments.

Table S3. Composition of modified SP and CL treatments for net uptake rate experiments in *Arabidopsis thaliana* plants.

Nutrients	SP (0.2 mM Cl^-)	CL (5.2 mM Cl^-)
**Na^+**	0.1	0.1
**K^+**	5.98	5.98
**Ca^{2+}**	1.58	1.58
**Mg^{2+}**	1.33	1.33
**Cl^-**	0.2	5.2
**NO_3^-**	5.0	5.0
**SO_4^-**	2.38	0.5
**PO_4^-**	1.88	0.63
**S+P**	4.26	1.13

Table S4. Relation of nutritional treatments for quantification of ion content in xylem sap secretion extracts in tobacco plants.

Nutrients (mM)	N:SP (3.5: 0.075 mM)	N:CL (3.5: 3.5 mM)
Na^{+}	0.1	0.1
K^{+}	4.23	4.23
Ca^{2+}	1.75	1.75
Mg^{2+}	2.75	2.75
Cl^{-}	0.075	3.5
NO_3^{-}	3.5	3.5
SO_4^{-}	3.16	1.14
PO_4^{-}	2.48	1.00
S+P	5.64	2.14

Nutritional treatments were a modification of the basal nutrient solution (BS) to obtain equimolar concentration of anions and cations in the N:CL treatment (3.5 mM Cl^{-} and 3.5 mM NO_3^{-}) and equimolar concentration of anions in the N:SP treatment of (3.5 mM NO_3^{-} and 5.6 mM PO_4^{-} + SO_4^{-}).
